# Enhancing maize drought and heat tolerance: single vs combined plant growth promoting rhizobacterial inoculation

**DOI:** 10.3389/fpls.2024.1480718

**Published:** 2024-12-10

**Authors:** Iviwe Notununu, Lucy Moleleki, Ashira Roopnarain, Rasheed Adeleke

**Affiliations:** ^1^ Department of Biochemistry, Genetics and Microbiology, Forestry and Agricultural Biotechnology Institute, University of Pretoria, Pretoria, South Africa; ^2^ Microbiology and Environmental Biotechnology Research Group, Agricultural Research Council - Soil, Climate and Water, Pretoria, South Africa; ^3^ Department of Life and Consumer Sciences, College of Agriculture and Environmental Sciences, University of South Africa, Roodepoort, South Africa; ^4^ Department of Environmental Sciences, College of Agriculture and Environmental Sciences, University of South Africa, Roodepoort, South Africa; ^5^ Unit for Environment Science and Management, North-West University, Potchefstroom, South Africa

**Keywords:** plant growth-promoting bacteria, drought and heat stress, plant-microbe interactions, biofertilizers, stress response genes, *Zea mays* L.

## Abstract

Maize (*Zea mays* L.), a key staple crop in Sub-Saharan Africa, is particularly vulnerable to concurrent drought and heat stress, which threatens crop yield and food security. Plant growth-promoting rhizobacteria (PGPR) have shown potential as biofertilizers to enhance plant resilience under such abiotic stresses. This study aimed to (1) identify PGPR isolates tolerant to drought and heat, (2) assess their capacity to mitigate the effects of these stresses on early maize growth, and (3) analyze maize gene expression changes associated with PGPR-induced tolerance. Rhizobacteria were isolated and screened for drought and heat tolerance, alongside key plant growth-promoting (PGP) traits, including phosphorus solubilization, nitrogen fixation, and indole acetic acid production. In vitro and pot trials evaluated the effects of selected isolates on maize growth under stress, using indicators such as shoot length, root and shoot biomass (wet and dry), and leaf water content. Quantitative reverse transcription PCR (qRT-PCR) was employed to profile maize stress response genes. The identified PGPR isolates included *Bacillus cereus* (11MN1), *Bacillus pseudomycoides* (21MN1B), *Lelliottia amnigena* (33MP1), and *Leclercia adecarboxylata* (36MP8). Greenhouse trials demonstrated that *L. amnigena* 33MP1, *L. adecarboxylata* 36MP8, and a mixed culture of isolates (11MN1, 21MN1B, 33MP1, 36MP8) effectively alleviated the adverse effects of concurrent drought and heat stress in maize. Notably, qRT-PCR analysis indicated that PGPR-induced tolerance may involve the modulation of stress response genes *CAT2* (catalase 2) and *DHN2* (dehydrin 2), which play roles in oxidative stress management and cellular protection. The PGPR isolates identified in this study represent promising bioinoculants for enhancing maize resilience under climate-induced stresses, offering a sustainable approach to improve maize productivity, conserve water, and reduce irrigation needs in drought-prone regions.

## 1 Introduction

Maize (*Zea mays* L.) is a major crop for human consumption and animal feed in Sub-Saharan Africa and is known for its susceptibility to drought and heat stresses (Badu-Apraku and Fakorede, 2017; Tesfaye et al., 2018; Nelimor et al., 2019). Under field conditions, high ambient temperatures often occur concurrently with water deficiency and are far more limiting to plant growth than the presence of these individual stresses alone (Farooq et al., 2012; Lipiec et al., 2013; Calvin et al., 2023). Previous simulation results indicate that climate change scenarios with rising temperatures and decreasing rainfall led to average maize yield reductions of 21%, 33%, and 50% with temperature increases of 1°C, 2°C, and 4°C above 30°C, respectively. In comparison, temperature-only scenarios caused yield reductions of 11%, 21%, and 41% for the same temperature increases (Tesfaye et al., 2018). Similarly, Meseka and colleagues found that while drought stress reduced maize grain yield by 58%, the combination of drought and heat stress reduced maize grain yield by 77%, highlighting the detrimental effects of these two combined stresses (Meseka et al., 2018).

The interactive effects of drought and heat stresses are known to alter plant metabolism differently compared to single stress (Rizhsky et al., 2004; Pandey et al., 2015; Shahsavandi et al., 2018). For example, enhanced transpiration, which is typical of plants under heat stress to cool the leaf surface, is aggravated under the dual effects of drought and heat stress leading to more water loss and increased salt uptake (Rizhsky et al., 2002; Choudhury et al., 2017). Furthermore, the concurrent effects of drought and heat stress on osmolyte accumulation, enzymatic and non-enzymatic antioxidants, nutrient uptake, and photosynthetic components of maize are more detrimental than when exposed to singular stresses (Hussain et al., 2019). The accumulation of reactive oxygen species (ROS) and rate of lipid peroxidation are reportedly higher when maize is exposed to combined stress, and these inhibit the photosynthetic efficiency thus restricting growth (Hussain et al., 2019). Furthermore, the transcriptomic analysis of combined heat and drought stressed plants has revealed a combination of shared and unique transcriptomic changes (Notununu et al., 2022a). Thus, highlighting that the combination of drought and heat stress is unique and cannot simply be extrapolated from the effect of each stress imposed individually, implying that plants have unique responses when presented with combined stress (Danish and Zafar-ul-Hye, 2019). Therefore, novel strategies that enhance performance under the two stresses are crucial for maintaining maize production.

Plant-microbe interactions are thought to be key to the adaptation and survival of plants to abiotic stresses (Ali et al., 2014; Zafar-ul-Hye et al., 2014a; Park et al., 2017; Al-Turki et al., 2023; Chieb and Gachomo, 2023). Studies have shown that the inoculation of plants growing under these extreme conditions with plant growth-promoting rhizobacteria (PGPR) could improve the plants’ tolerance to stresses by directly or indirectly influencing their growth and morphology, thus enhancing their development and yield (Cohen et al., 2009; Kang et al., 2019). These microorganisms can achieve this feat because, like plants, they experience hydric and heat stress when water availability is low and temperatures are high. However, they have developed adaptive mechanisms to survive under these challenging conditions. This resilience enables them to support plants facing similar stresses. This is attributed to PGPRsensing such changes in soil and quickly adapting in a way that is also beneficial for plants (García et al., 2017). The various mechanisms utilized by PGPR that help plants gain resistance to drought and heat stresses include modification in antioxidant defense, phytohormonal levels, production of exopolysaccharides (EPS), heat shock proteins (HSPs), dehydrins, volatile organic compounds (VOCs), and metabolic adjustments (Kaushal and Wani, 2016; Nikhil et al., 2023).

Therefore, the current study aimed to screen and identify bacterial isolates with plant growth-promoting attributes and tolerance to drought and heat stress. Furthermore, the study evaluated the ability of selected drought and heat-tolerant PGPR isolates at mitigating the effects of dual drought and heat stress on the early growth of maize, as well as unravel selected innate maize drought and heat-stress response genes, which actively participate in PGPR-induced tolerance.

## 2 Materials and methods

### 2.1 Microbial screening for plant growth promoting characteristics

Soil samples were collected from the rhizosphere of maize in Sheepmoor village, Msukaligwa municipality located in Gert Sibande District Municipality, Mpumalanga province, South Africa, coordinate 26’’ 45’18’’ S, 30’ 13’55’’ E (**Supplementary Figure S1**) (Tsipinana, 2019). Bacterial isolates obtained were screened for plant growth-promoting attributes in a previous study (Tsipinana, 2019). Isolates found to exhibit high plant growth promoting (PGP) abilities were screened for drought and heat tolerance.

#### 2.1.1 Screening of isolates for phosphorus solubilizing characteristics

Briefly, the evaluation of phosphorus-solubilizing microorganisms was conducted according to the methods outlined by Paul and Sinha (2017) and Sharma and Gobi (2016). One gram of rhizosphere soil was transferred to 9 ml of sterile distilled water and vigorously shaken. The resulting suspension was serially diluted (10^-1^-10^-6^), and 100 μL aliquots of the 10^-6^ dilution were plated on Pikovskaya’s Agar medium containing insoluble tricalcium phosphate. The plates were then incubated at 30°C for 7 days. Any colonies exhibiting a halo zone were selected and sub-cultured on Pikovskaya’s (PVK) agar medium to obtain pure isolates. The pure isolates were then transferred to a nutrient broth and incubated at 30°C for 24 hrs. Subsequently, three wells (equidistant from each other and situated near the center of the plates) were then created on the plates containing Pikovskaya’s Agar. Equal amounts of the pure cultures in the broth were transferred to each well, and the plates were incubated at 30°C for 7 days. The diameter of the well and halo were then measured. The solubilizing index (SI) was calculated as follows: *SI = Solubilisation diameter + Colony diameter ÷ Colony diameter :*

#### 2.1.2 Screening of isolates for nitrogen fixation

The presence of nitrogen-fixing microorganisms was assessed by a method outlined by Park et al. (2005). To begin, 1 gram of rhizosphere soil was mixed with 9 ml of sterile distilled water and vigorously shaken. Next, serial dilutions were prepared, and 100 μL aliquots of the 10^-6^ were plated on Burk’s N-free medium. The plates were then incubated at 30°C for 7 days. The appearance of colonies on the medium indicated the ability to fix atmospheric nitrogen. Any morphologically different colonies that appeared on the medium were isolated and sub-cultured for further study.

#### 2.1.3 Screening of isolates for indole acetic acid production

The process for determining the production of Indole Acetic Acid (IAA) hormone by nitrogen-fixing and phosphate-solubilizing microorganisms was outlined by Mohite (2013). To start, the isolates were grown in 10 mL of nutrient broth at 30°C for 24 hours on a shaker set at 200 rpm. Next, 2 mL of the microbial suspension was spun at 10,000 rpm for 10 minutes at 4°C, and the supernatant was then transferred to a test tube. Following this, 2 mL of Salkowsky’s reagent (a solution of 150 mL concentrated H2SO4, 250 mL distilled water, and 7.5 mL of 0.5 M FeCl3) was added and incubated at room temperature in the dark for 30 minutes. The absorbance of the solution was then measured using a spectrophotometer at 540 nm. A standard curve of known IAA concentrations was established, and different concentrations of IAA solution (0, 5, 10, 20, 50, and 100 μg/mL) and used to calculate the concentration of IAA in the samples.

### 2.2 Microbial screening for drought stress tolerance

Bacteria with drought tolerance were screened using a 40% polyethylene glycol (PEG) 6000 solution in tryptic soy broth (TSB). The resulting osmotic potential of this solution is approximately -2.70 MPa (Ali et al., 2014). Single colonies of test isolates were used to inoculate 10 ml TSB and grown for 24 h at 32 °C on a shaking incubator rotating (IncoShake, Labotec, Johannesburg, South Africa) at 120 rpm and the growth was estimated by measuring the optical density at 600 nm using a DR 5000™ UV-Vis spectrophotometer (Hach, Loveland, United States) (Bashan and Levanony, 1990; Zafar-Ul-Hye et al., 2014b). Bacterial isolates were considered drought stress-tolerant if an OD_600_ greater than 0.400 was recorded. All tests were conducted in biological four replicates.

### 2.3 Microbial screening for heat stress tolerance

Heat-tolerant isolates were screened by adding an appropriate volume of a previously grown bacterial suspension to fresh TSB, achieving a uniform cell density with an OD_600_ of 0.1 in a final volume of 10 mL (Praveen Kumar et al., 2014). The bacterial solutions were then incubated at 42°C in a shaker at 120 rpm for 24 h, and the bacterial growth was estimated by measuring the optical density at 600 nm using a DR 5000™ UV-Vis spectrophotometer (Hach, United States). Bacterial isolates were considered heat stress-tolerant if an OD_600_ greater than 0.400 was recorded. All tests were conducted in four biological replicates.

### 2.4 PCR and phylogenetic characterization of both drought and heat tolerant isolates

Bacterial genomic DNA was isolated using the Quick-DNA Fecal/Soil microbe kit (Zymo Research, Irvine, CA, United States) according to the manufacturer’s protocol. Polymerase Chain Reaction (PCR) for amplification of the 16S rDNA gene was conducted using 50 ng of genomic DNA and universal forward 341F (5’-CCTACGGGAGGCAGCAG-3’) and reverse 907R (5’-CCGTCAATTCCTTTRAGTTT-3’) primers. Briefly, 1 µL of the sample was added to 19 μL of Luna Universal qPCR Master Mix and primers (concentration of 0.25 μM). The PCR cycling conditions were as follows: After denaturation at 95°C for 5 min, 35 rounds of temperature cycling (95°C for 1 min, 55°C for 1 min and 72°C for 3 min) were followed by incubation at 72°C for 7 min. The PCR product purity was confirmed by electrophoresis on 1% agarose gel. The PCR products were sent for Sanger sequencing (Inqaba Biotec, South Africa). DNA sequences were edited using Bioedit sequence alignment editor (Hall, 1999) and compared to known sequences using NCBI’s nucleotide Basic Local Alignment Search Tool (BLAST) (Altschul et al., 1990). Molecular Evolutionary Genetics Analysis (MEGA) was used for statistical analysis of molecular evolution and for constructing phylogenetic trees.

### 2.5 Surface sterilization and germination of maize seeds for *in vitro* experiments

The seeds used for the study were the commercially available “Zama Star’ obtained from Starke Ayres (Pretoria, South Africa). Maize seeds showing no disease symptoms were used for the current experiment. Maize seeds were surface sterilized to remove any microbes on the surface using 70% (v/v) ethanol and agitated using a shaker for 15 min at 120 rpm. Then the seeds were disinfected with 3.5 (v/v) % sodium hypochlorite for another 15 min. The seeds were then rinsed three times using sterile distilled water (Akinyosoye et al., 2015). The maize seeds were germinated between two filter papers in Petri dishes filled with 10 ml of sterile distilled water. A total of 10 seeds were placed per Petri dish facing up. The Petri dishes containing surface-sterilized seeds were incubated in the dark at 27°C for 7 days. Germinated maize seeds with a root of 1-2 cm were chosen and used in the subsequent steps.

### 2.6 Inoculation of maize seedlings for *in vitro* experiments

The germinated maize seedlings were inoculated with the bacteria strains following the method described by Niu and Kolter (Niu and Kolter, 2018). Each bacterial isolate was streaked on tryptic soy agar (TSA) plates and incubated for 24 h at 32°C. Afterward, a single colony was inoculated in 10 ml of TSB and incubated in a shaker rotating at 120 rpm at 32°C overnight. Following incubation, the bacteria cells were placed on ice and then collected in 2.0 ml Eppendorf tubes by centrifuging at 2,940 × g for 10 min. The cells were resuspended in 0.85% NaCl saline solution. Approximately 10 surface-sterilized and germinated maize seeds with primary roots of 1-2 cm were soaked in 30 ml (~10^8^ CFU/ml) of the bacterial suspensions in Petri dishes at room temperature for 1 h. Another 10 surface-sterilized and germinated seeds were soaked in 0.85% NaCl saline solution for 1 h and used as a control. Following the 1 h, the maize seedlings were transferred onto 25% strength Murashige and Skoog (MS) media (supplemented with 5 g/L sucrose, 8 g/L bacteriological agar and adjusted to pH 5.8) infused with 20% PEG 6000 in magenta boxes (van der Weele et al., 2000; Verslues and Bray, 2004). The magenta boxes were incubated in a growth room with a 16 h/8 h light/dark cycle, at 32°C/28°C day and night temperatures at a relative humidity of 50% for 14 days. Thereafter, shoot length, fresh and dry weight, and moisture content were measured.

### 2.7 Preparation of bacteria inoculum and inoculation of maize seeds for pot-plant experiments

Bacteria isolates that previously showed the potential to alleviate the detrimental effects of dual drought and heat stress on maize *in vitro* were streaked on TSA plates and incubated at 32°C for 24 h. Afterward, a single colony of each strain was inoculated in 100 ml of tryptic soy broth (TSB) and shaken at 120 rpm at 32°C overnight. The cells were collected in 50 ml tubes by centrifuging at 2,940 × g for 10 min at 4°C. The cells were resuspended in 40 ml 0.85% saline solution. Viable cells were estimated using the dilution series and direct colony count method. Subsequently, the bacterial suspensions were diluted to ~10^8^ CFU/ml with 0.85% saline solution. For multiple strain/mixture inoculum, the bacteria suspensions were mixed in equal volumes to give a 40 ml solution.

The maize seeds were surface sterilized as described above. A total of 30 seeds were then inoculated with bacteria by soaking in 40 ml of ~10^8^ CFU/ml concentration of each bacterial solution for 1 h. An additional 30 surface-sterilized seeds were soaked in 0.85% saline solution for 1 h and used as a control.

### 2.8 Pot-plant experiments

A total of 1.6 kg of heat-treated (80 °C for 1 hr) (bark and peat-based) potting mix was weighed into pots and pre-irrigated with distilled water and allowed to drain for 2h. Seeds previously soaked in bacteria solutions were planted 5 cm deep. Seven seeds were planted per pot. Under no stress control conditions, the seedlings were grown under greenhouse conditions at 23/25°C for 32 days. The water holding capacity was maintained at 80% by watering, when necessary, with distilled water. Under dual drought and heat-stressed conditions, the seedlings were grown for 32 days at 32/28°C, with the water holding capacity maintained at 40% by watering, when necessary, with distilled water. Pots were fertilized with 50 ml Murashige and Skoog (MS) twice during the growth period; at planting and 15 days after planting. Plant growth parameters were then assessed namely, plant height, shoot and root dry mass (Wood and Roper, 2000) as well as the leaf relative water content (RWC) (Teulat et al., 2003; Arndt et al., 2015). Leaf samples for qPCR were harvested after 32 days and stored at -80°C.

### 2.9 Plant material and RNA preparation

Maize leaves stored at -80°C were ground to fine powder in liquid nitrogen. Total RNA was extracted using the Trizol Reagent following manufacturer instructions (Thermo Fisher Scientific, America) and quantified using the NanoDrop (Thermo Fisher Scientific, USA). The quality and integrity of extracted RNA was checked by running it on a 1% (w/v) agarose gel containing 0.5% (w/v) sodium chloride (Aranda et al., 2012). The RNA was stored at -80°C until use.

### 2.10 RT-qPCR

Complementary DNA was synthesized from 50 ng of total RNA using the LunaScript^®^ RT SuperMix Kit (New England Biolabs, UK) as instructed by the manufacturer. Quantitative real-time PCR was conducted in 96-well plates using Luna Universal qPCR Master Mix in the CFX96 Touch Deep Well Real-Time PCR System (Bio-Rad, USA). Briefly, 1 µL of the sample was added to 19 μL of Luna Universal qPCR Master Mix and primers (concentration of 0.25 μM). The cycling conditions were as follows: initial denaturation at 95°C for 1 min followed by 39 cycles of denaturation at 95°C for 15 s and primer annealing at 65°C for 1 min. Each treatment had 3 biological replicates and two technical replicates. Afterward, the melting curves ranging from 60°C to 95°C were evaluated in each reaction to check the specificity of the amplicons. The samples were normalized against beta Tubulin (*β-TUB*) and Elongation factor 1 alpha (*EF1a*) as the reference genes and the control uninoculated samples were used as calibrators (Lin et al., 2014). The selected stress response genes of interest included, Dehydrin 2 (*DHN2*), Heat Shock Protein 70 (*HSP70*) and Catalase 2 (*CAT2*) (Capelle et al., 2010). The comparative CT (ΔΔ^ct^) method was used to measure relative expression (Livak and Schmittgen, 2001). To check whether RT-qPCR results were statistically different when comparing inoculated samples to uninoculated control samples, the two-tailed Student’s *t*-test (unequal variances) was used (*P* < 0.05). Some primers were obtained from previous studies, and some were designed using Primer3Plus (http://primer3plus.com/cgi-bin/dev/primer3plus.cgi) and are listed in **Supplementary Table S1**. Primer specificity was checked using NCBI’s Primer-BLAST (https://www.ncbi.nlm.nih.gov/tools/primer-blast/).

### 2.11 Statistical analyses

To check whether the measured plant growth parameters were statistically different between treatments, the two-tailed Student’s *t*-test (unequal variances) at *P* < 0.05 confidence level was conducted using R studio. Each treatment was replicated four times, and the data were expressed as mean ± SD. Different letters were used to depict treatments with statistical differences, whereas the same letters indicated that there were no statistical differences.

## 3 Results

### 3.1 Isolates exhibiting drought and heat tolerance

Sixty one isolates previously shown to have PGP abilities were screened for the ability to grow adequately under reduced osmotic pressure and elevated temperatures. Out of the 61 isolates screened with plant growth-promoting (PGP) attributes, more than 90% demonstrated efficient growth at a temperature of 42°C (OD_600_ greater than 0.40 was recorded) (**Supplementary Table S2**). A total of six (less than 10%) had an OD_600_ measurement that was below 0.40 (**Table 1**).

**Table 1:** PGPR with high tolerance to drought and heat stress and their respective plant growth-promoting properties.

Isolate ID	N-Fixation	P Solubilization	IAA Conc ug/ml)	Heat Tolerance (42°C stress) (OD)	Drought Tolerance (40 % PEG 6000) (OD)
32MN1B	**	‐‐	20.75	1.1	0.5
14MN3B	**	‐‐	13.77	1.05	1.15
14MN5A	**	‐‐	16.85	1.12	0.49
21MN2B	**	‐‐	11.04	1.02	0.45
11MN3	**	‐‐	28.77	0.93	0.42
21MN1B	**	‐‐	14.99	0.94	0.45
11MN1	**	‐‐	12.78	1.07	0.53
11MN2	**	‐‐	13.19	1.14	0.5
31MN1B	**	‐‐	10.46	0.66	0.5
33MP1	‐‐	**	15.81	0.87	0.53
34MP2	‐‐	**	13.77	0.86	0.48
36MP8	‐‐	**	13.54	0.64	0.96

**Indicate positivity for characteristic.

‐‐Indicates negativity for characteristic.

Conversely, twelve isolates (11MN3, 14MN3B, 36MP8, 34MP2, 33MP1, 11MN1, 11MN2, 32MN1B, 31MN1B, 14MN5A, 21MN1B, 21MN2B) achieved an OD_600_ greater than 0.40 under TSB supplemented with 40% PEG 6000 showing tolerance (**Table 1**; **Supplementary Table S2**). All 12 of these bacterial isolates also displayed tolerance to heat stress, thus making a total of 12 isolates that exhibited the potential to tolerate both drought and heat stress (**Table 1**). From these isolates, nine exhibited nitrogen-fixing attributes, whereas only three showed P-solubilizing capabilities (**Table 1**) (Tsipinana, 2019; Notununu et al., 2022a).

### 3.2 Identification of isolates

Isolates that demonstrated capabilities to tolerate both drought and heat stress were further characterized using the 16S rRNA gene for identification (**Supplementary Table S3**). Isolates 11MN1, 11MN2, 11MN3, 14MN5A, 21MN2, 31MN1, and 32MN1B had 100% sequence similarity with *Bacillus cereus* strain 24195 according to NCBI’S nucleotide BLAST. Isolate 14MN3A had a 99% sequence similarity with *Acinetobacter* sp. DSM30007. Isolate 21MN1B had a 100% sequence similarity with *B. pseudomycoides* strain MF-68. Isolate 33MP1 showed 99% sequence similarity with *Lelliottia amnigena* strain NCTC12124 a type of *Enterobacteriaceae*. Isolates 34MP2 and 36MP8 showed 99 and 100% sequence similarity with *Leclercia adecarboxylata*, respectively that also belong to the *Enterobacteriaceae* group.

**Figure 1:**
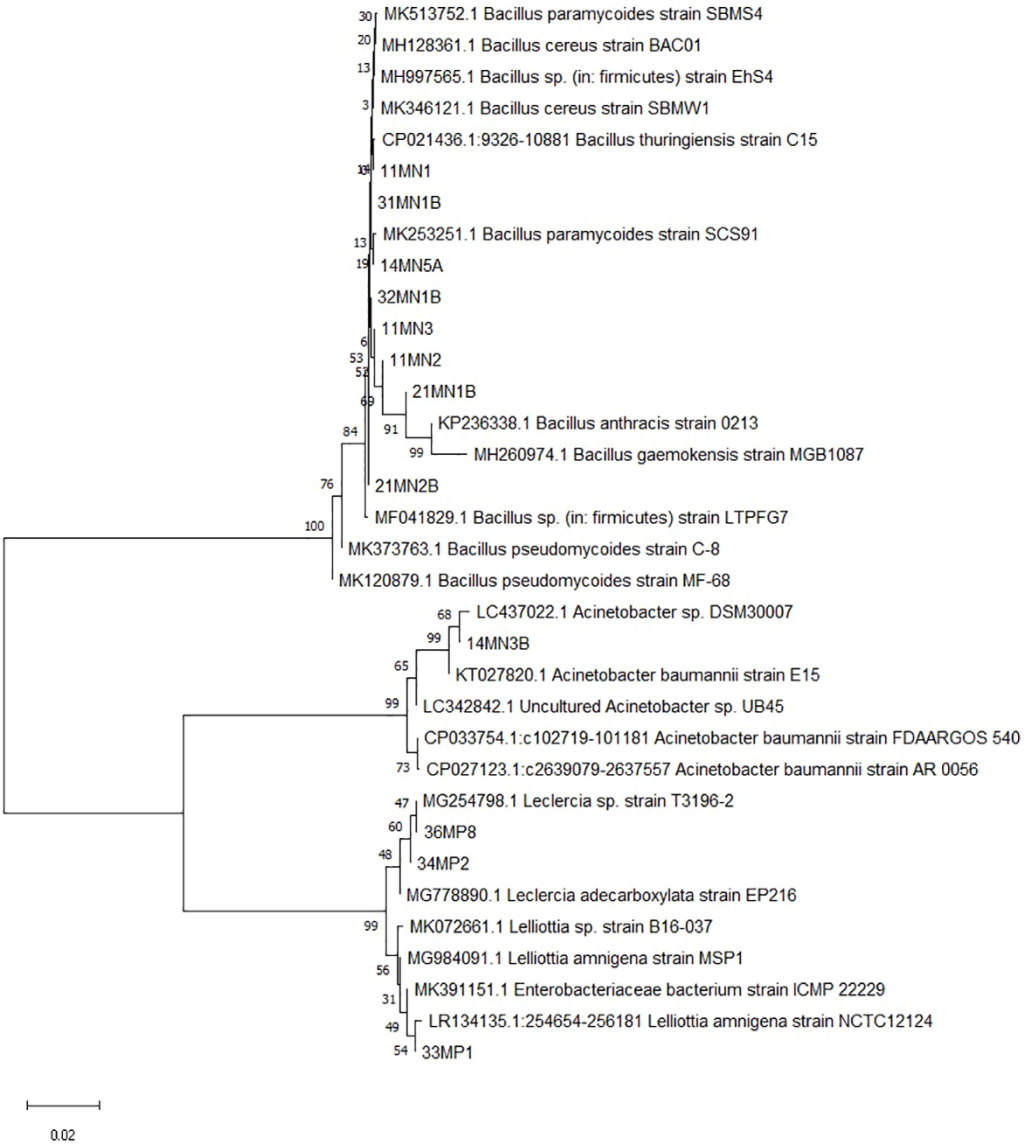
Evolutionary relationships of taxa using the Neighbour-Joining method based on the 16S rRNA gene. The bootstrap consensus tree inferred from 1000 replicates was taken to represent the evolutionary history of the taxa analysed. The percentage of replicate trees in which the associated taxa clustered together in the bootstrap test (1000 replicates) are shown next to the branches. The evolutionary distances were computed using the Maximum Composite Likelihood method.

The phylogenetic tree in **Figure 1** groups together several isolates, namely 32MN1B, 14MN5A, 21MN2B, 11MN3, 11MN1, 31MN1B, and 11MN2, when using the Neighbor-Joining method based on the 16S rRNA gene. These isolates indicate a close evolutionary relationship with *B. cereus.* Isolate 21MN1B appears to have a similar evolutionary background to *B. pseudomycoides*, while isolate 14MN3A seems to be closely related to *A. baumanni*. Additionally, isolates 34MP2, 33MP1, and 36MP8 cluster together, suggesting a shared evolutionary background similar to known *Enterobacteriaceae*, *L. adecarboxylata*, and *Lelliotta* sp.

### 3.3 Influence of single inoculum on maize growth under simultaneous drought and heat stress *in vitro*

**Figure 2:**
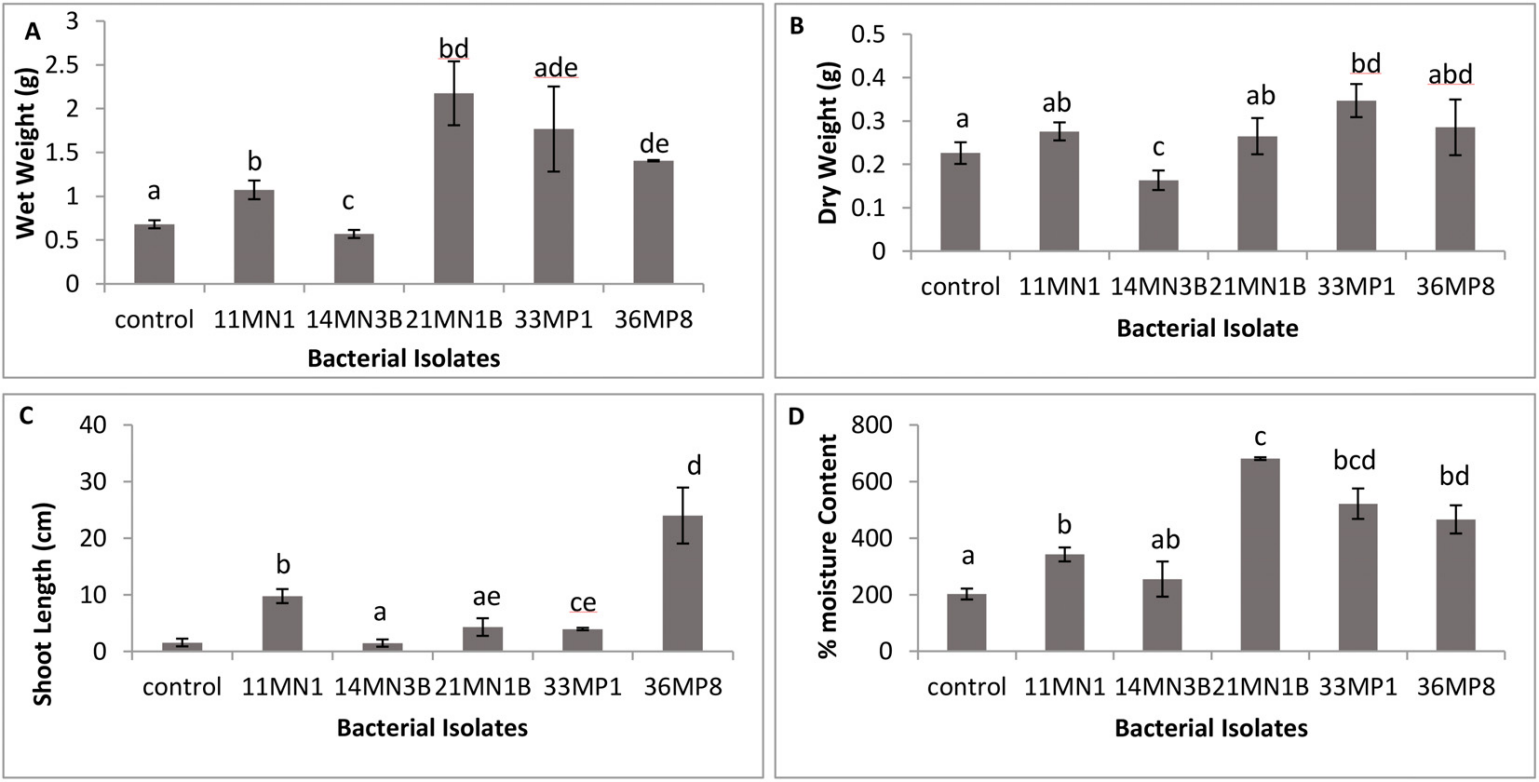
The effect of selected bacteria isolates with PGPR characteristics on maize whole wet and dry weight, shoot length and moisture content *in vitro* under simulated drought and heat stress conditions. Drought was simulated by adding 20% PEG 6000 and heat stress was simulated by incubation plants at 32/28°C day and night temperatures. Student T test; p < 0.05; different letters indicate significant differences. **(A)** Wet weight, **(B)** Dry weight, **(C)** shoot length, **(D)** moisture content.

**Figure 3:**
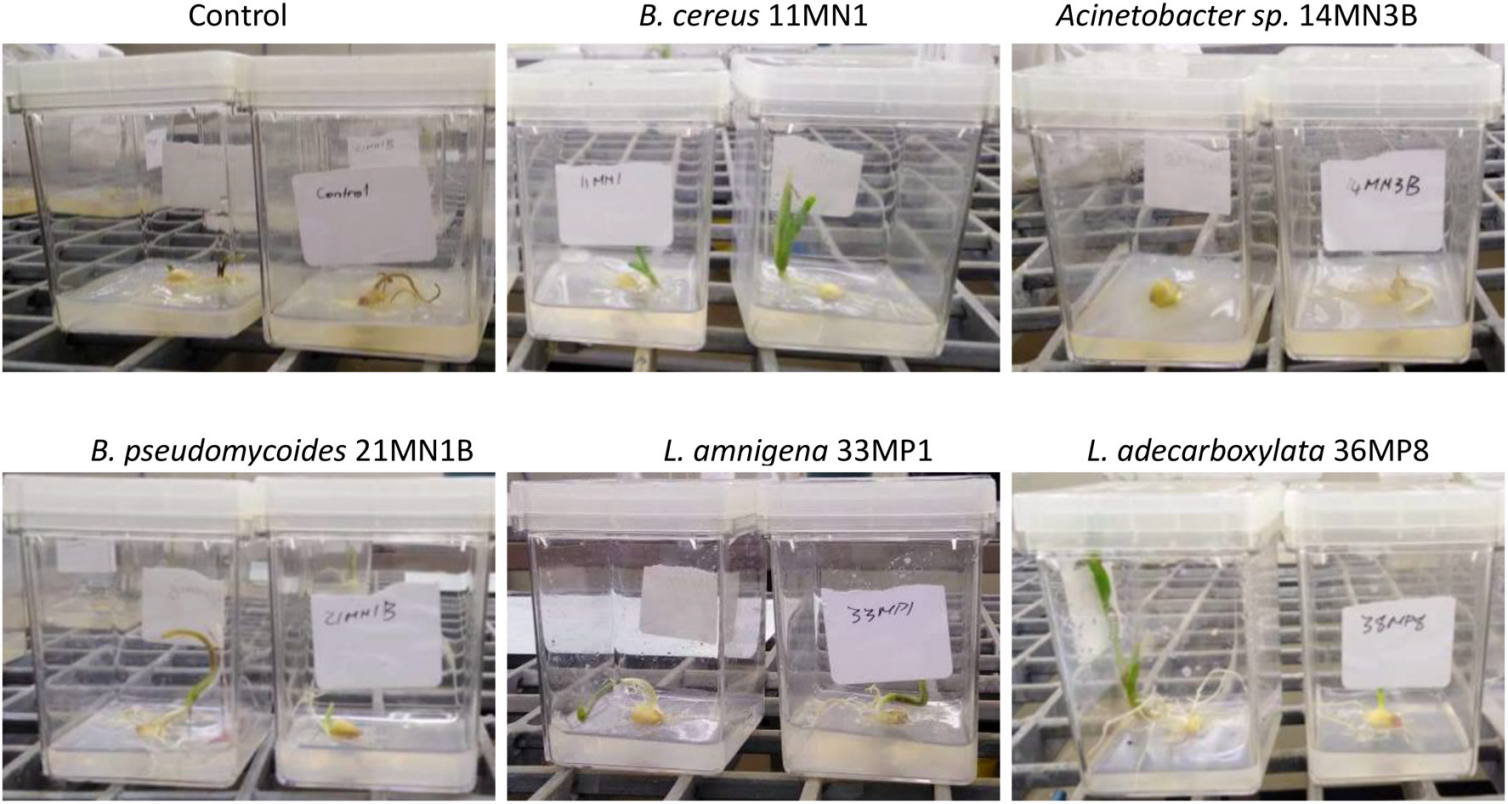
Images illustrating plants that have been exposed to specific bacterial isolates while experiencing both drought and heat stress.

The effects of drought and heat tolerant PGPR strains on the growth of maize seedlings was investigated *in vitro*. All isolates except for 14MN3B improved all the measured plant growth parameters of maize seedlings *in vitro* under the combined drought and heat stress compared to the untreated control plants (**Figures 2**, **3**). Isolate 11MN1, 21MN1B, and 36MP8 significantly (p <0.05) improved the fresh weight of maize seedlings by 48.52, 220.59 and 107.35% respectively, compared to the control (**Figure 2A**). Only isolate 33MP1 (53.53%) significantly improved maize dry weight compared to the control (**Figure 2B**). Inoculation of maize plants with isolate 11MN1, 33MP1 and 36MP8 significantly improved the shoot length (P <0.05) of the maize seedlings (**Figure 2C**). Maize moisture content was significantly improved by 43.75, 258.70, 103.6, and 95.48% for 11MN1, 21MN1B, 33MP1, and 36MP8, respectively compared to the control plants (**Figure 2D**). Maize seedlings inoculated with isolate 21MN1B resulted in the highest moisture content.

### 3.4 Influence of combined inoculum on maize growth under simultaneous drought and heat stress *in vitro*

**Figure 4:**
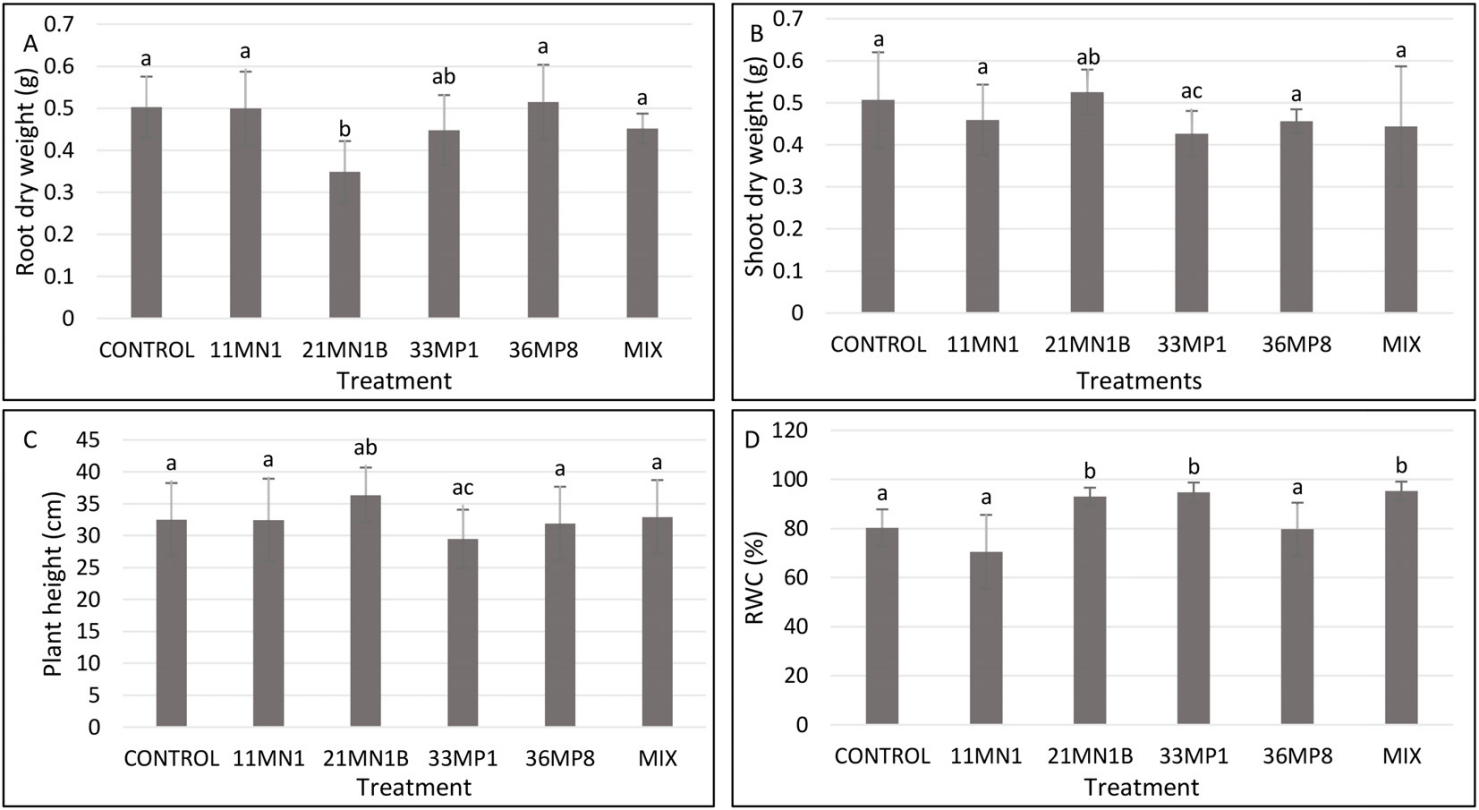
Effects of PGPR isolates on maize plant growth parameters cultivated under greenhouse conditions, with 80% soil WHC and temperature of 25/23°C. significant differences between treatments were conducted using the student’s t-Test p < 0.05. Different letters distinguish between statistically different treatments whereas same letters suggest no statistical differences between treatments. **(A)** Root dry weight, **(B)** shoot dry weight, **(C)** Shoot length, **(D)** relative water content (RWC).

Under unstressed conditions, the soil water holding capacity was maintained at 80% and the temperature at 25/23°C. No significant enhancement in root dry biomass, shoot dry weight, and plant height were observed in plants inoculated with the isolates (**Figures 4A–C**; **Supplementary Figure S2**). However, plants inoculated with 21MN1B, 33MP1 and the mixture of all isolates showed significantly increased relative water content (**Figure 4D**). Maize plants inoculated with 21MN1B, 33MP1, and the mixture had a significantly improved RWC (P <0.05) by 15.8, 17.9, and 18.6% compared to the untreated control plants, respectively (**Figure 4D**).

**Figure 5:**
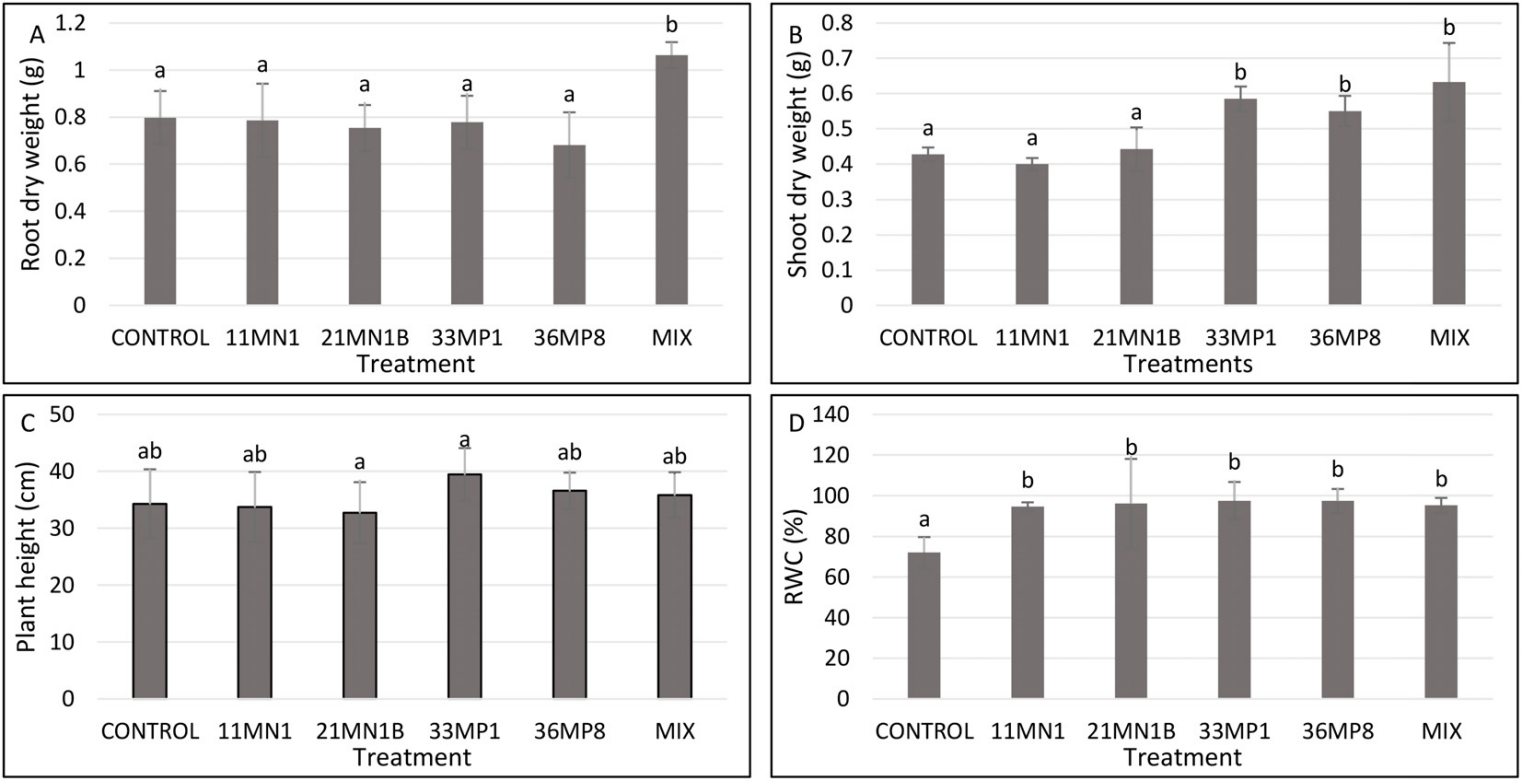
Effects of PGPR isolates on maize plant growth parameters cultivated under greenhouse conditions, with 40% soil WHC and temperature of 32/28°C. Significant differences between treatments were conducted using the student’s t-Test p < 0.05. Different letters distinguish between statistically different treatments whereas same letters suggest no statistical differences between treatments. **(A)** Root dry weight, **(B)** shoot dry weight, **(C)** Shoot length, **(D)** relative water content (RWC).

Under stress conditions where the soil’s water holding capacity is reduced to 40%, and with high temperatures reaching 32/28°C, plants that were treated with bacterial isolates displayed enhanced growth in one or more of the measured plant growth parameters when compared to plants that were not treated with these isolates. (**Figures 5A–D**; **Supplementary Figure S3**).

According to the results, the root dry weight was improved by 33% in plants inoculated with a mixture of all isolates (11MN1, 21MN1B, 33MP1, 36MP8) (P <0.05) compared to the untreated control (**Figure 5A**). Isolate 33MP1, 36MP8, and the mixture significantly improved shoot dry weight (P <0.05) by 36.9, 28.7, and 47.9% compared to the untreated control, respectively (**Figure 5B**). No significant differences were observed in plant height between all treatments and the untreated control (**Figure 5C**). Conversely, the isolates significantly improved leaf RWC Isolates 11MN1, 21MN1B, 33MP1, 36MP8, and the mixture increased maize leaf RWC (p< 0.05) by 31.2, 33.3, 35.4, 35.1, and 32.0% compared to control, respectively, under stressed conditions (**Figure 5D**).

### 3.5 Gene expressions of stress response genes of maize leaves

**Figure 6:**
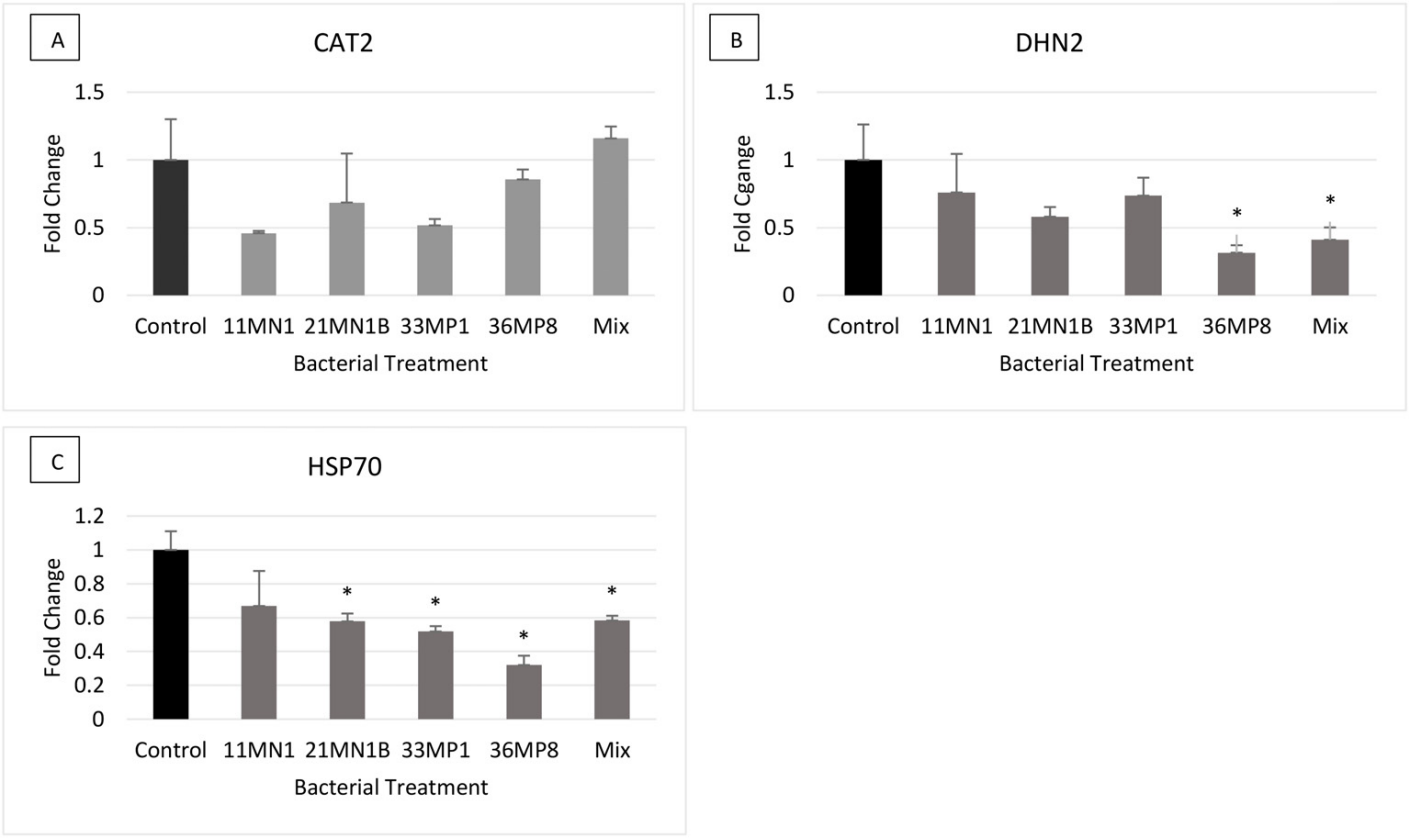
The expression of stress response genes on PGPR inoculated maize plants grown under greenhouse conditions under ambient no stress conditions (soil WHC 80% and temperature of 25/23°C). The genes of interest include Dehydrin 2 (DHN2), Heat Shock Protein 70 (HSP70) and Catalase 2 (CAT2). Tubulin beta (β-TUB) and Elongation factor 1 alpha (EF1a) were used as the reference genes. Error bars represent the SEM of fold change determined using 2^-ΔΔCT ± SEM^ (n = 3). Significant differences between control/calibrator sample and inoculated samples were conducted using the student’s t-Test, p < 0.05. The Asterix (*) distinguishes treatments that are significantly different from controls. Gene expression profiles are depicted as follows: **(A)** CAT2, **(B)** DHN2, **(C)** HSP70.

Under no stress conditions, no significant differences (p< 0.05) were observed in the relative expression of the *CAT2* gene in all treatments, compared to the untreated control (**Figure 6**). The *DHN2* gene was statistically differentially expressed on maize plants inoculated with 36MP8 and the mixture, with downregulation of 0.68 and 0.59-fold respectively. The *HSP70* gene was significantly downregulated in maize plants inoculated with 21MN1B (0.43-fold), 33MP1 (0.48-fold), 36MP8 (0.68-fold), and the mixture (0.42-fold) relative the untreated control plants.

**Figure 7:**
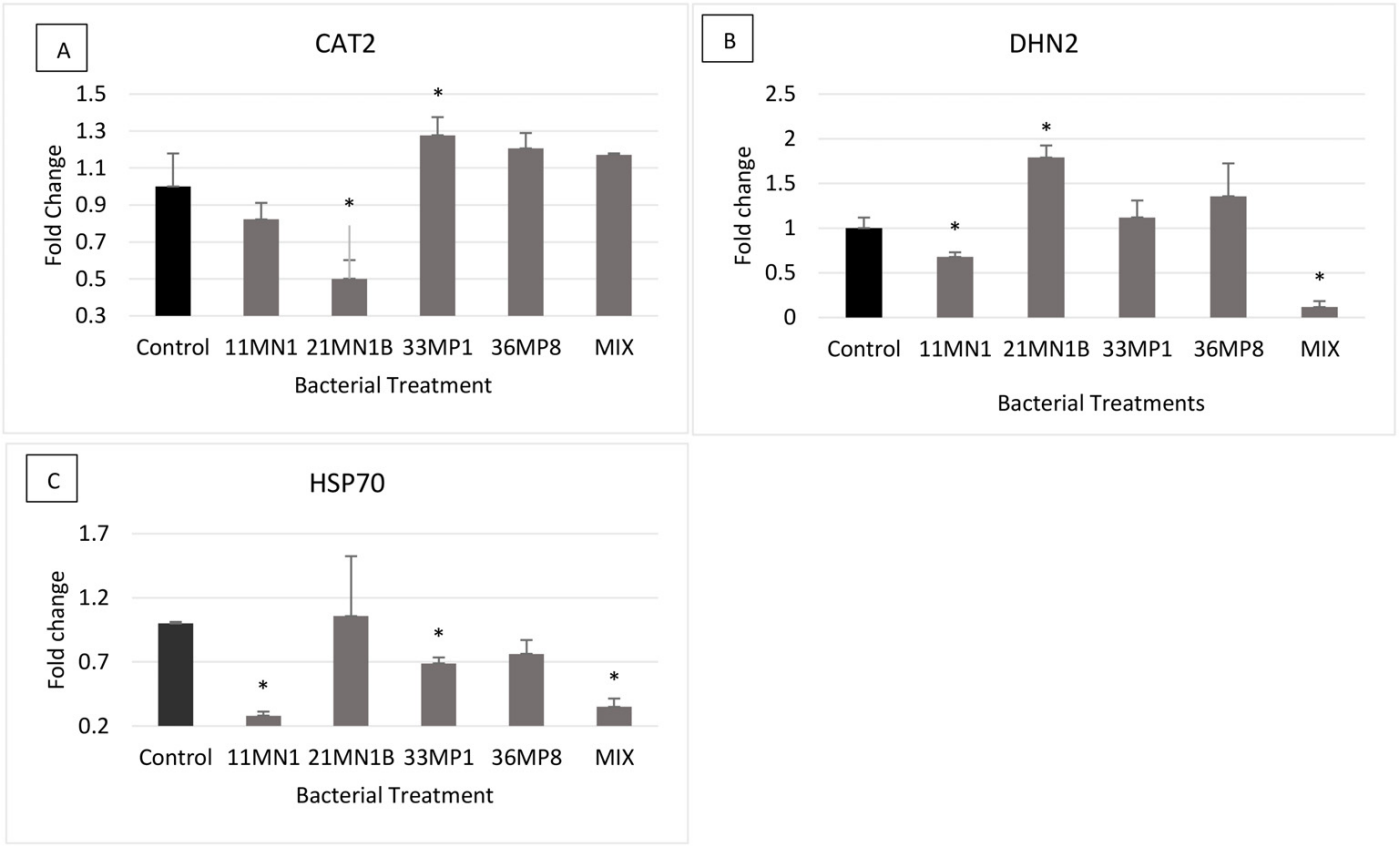
The expression of stress response genes on PGPR inoculated maize plants grown under greenhouse conditions under concurrent drought and heat stress conditions (soil WHC 40% and temperature of 32/28°C). The genes of interest include Dehydrin 2 (DHN2), Heat Shock Protein 70 (HSP70) and Catalase 2 (CAT2). Tubulin beta (β-TUB) and Elongation factor 1 alpha (EF1a) were used as the reference genes. Error bars represent the SEM of fold change determined using 2^-ΔΔCT ± SEM^ (n = 3). Significant differences between control sample and inoculated samples were conducted using the student’s t-Test, p < 0.05. The Asterix (*) distinguishes treatments that are significantly different from controls. Gene expression profiles are depicted as follows: **(A)** CAT2, **(B)** DHN2, **(C)** HSP70.

Under stressed conditions, only plants inoculated with 21MN1B showed statistically differential expression of the *CAT2* gene compared to the control by 0.5-fold downregulation (**Figure 7A**). The *DHN2* gene was significantly downregulated in maize plants inoculated with isolate 11MN1 (downregulated by 0.33-fold) and the mixture (downregulated by 0.88-fold) compared to the untreated control plants. However, only plants inoculated with 21MN1B had the *DHN2* gene upregulated (by 0.78-fold) relative to the untreated control plants (**Figure 7B**). The *HSP70* gene was statistically downregulated by 0.72, 0.32, and 0.65-fold for plants inoculated with 11MN1, 33MP1, and the mixture, respectively compared to the untreated control plants (**Figure 7C**).

## 4 Discussion

Plants are constantly exposed to abiotic stresses, such as drought and heat-stresses that cause serious problems affecting plant growth and development. Moreover, under field conditions, these abiotic stresses commonly occur more frequently in combination than as isolated incidents. PGPR inoculation of plants exposed to these abiotic stresses has been shown to mitigate the resultant detrimental effects (Notununu et al., 2022a). Furthermore, PGPR inoculated as a consortium as opposed to single isolates, has been shown to infer superior abiotic stress alleviating attributes. As such, the current study screened several previously isolated PGPR for their ability to tolerate drought and heat stress and evaluated the potential of these bacterial isolates to mitigate stress on maize plants as single isolates and as a combined mixture under *in vitro* and under greenhouse conditions.

The study found 12 PGPR isolates with the potential to tolerate drought and heat stresses. Phylogenetic analysis of the 16S rRNA gene sequence revealed that the selected isolates belonged to four genera: *Bacillus, Lelliottia, Acinetobacter*, and *Leclercia*. One PGPR isolate was utilized from each genus in subsequent studies, the following isolates were chosen: *B. cereus* 11MN1, *Acinetobacter* sp. 14MN3A, *B. pseudomyciodes* 21MN1B, *L. amnigena* 33MP1, and *L. adecarboxylata* 36MP8. These isolates were selected for their superior ability to tolerate drought and heat stress, as well as their enhanced plant growth-promoting properties compared to other strains of the same species. The selection of species from different genera increases the diversity of microorganisms, which in turn leads to greater genetic diversity and functional capabilities (Molina-Romero et al., 2017; Thomloudi et al., 2019). This diversity is crucial for improving plant tolerance to multiple stresses and optimizing their growth and productivity under challenging conditions. It was imperative for these isolates to exhibit exceptional resilience to drought and heat stress, as research has demonstrated that inoculating plants with tolerant PGPR significantly enhances their response to abiotic stress (García et al., 2017). These isolates could adequately grow under artificially induced drought and heat stress in the current study. The ability to survive drought and heat stress indicated that these isolates could potentially grow and infer beneficial effects in plants subjected to the same abiotic stresses. *Bacillus cereus* CECT 148 T was found to synthesize and accumulate the β-amino acid-type compatible solute Nε-acetyl-β-lysine (NeABL) (Triadó-Margarit et al., 2011). Additionally, *Bacillus cereus* G2 enhanced proline and glycine betaine metabolism in *Glycyrrhiza uralensis* under salt-induced osmotic stress, which increased the plant’s relative water content (Peng et al., 2023). Furthermore, inoculation with *Bacillus mycoides* PM35 in salinity-induced osmotic stressed maize plants resulted in higher levels of total soluble sugars and protein content, further supporting the plant’s stress tolerance and growth under adverse conditions (Ali et al., 2022). These compounds are responsible for osmotic adjustment, the detoxification of ROS, and the stability of the quaternary structure of proteins under water deficit conditions (Khan et al., 2019). PGPR can release these compounds extracellularly for assimilation by plants, thereby enhancing plant resistance to abiotic stress factors. Moreover, the previously demonstrated PGPR characteristics of these isolates, including nitrogen fixation, indole acetic acid and siderophore production, as well as phosphorus solubilization (Tsipinana, 2019; Notununu et al., 2022b), could continue to contribute to improved plant growth even when subjected to both drought and heat stress simultaneously. It has been shown that benefits inferred to plants by PGPR are reportedly not only attributed to a single plant growth-promoting characteristic, but multiple plant growth-promoting mechanisms are involved. This is explained by the “Multiple Mechanism Theory” formulated by Bashan and Levanony (1990), which assumes that several factors influence the successful association of these PGPR with plants (Zeffa et al., 2019). These factors include the bacteria’s ability to produce plant hormones, enhance nutrient availability, and improve stress tolerance in plants. The theory suggests that the cumulative effect of these mechanisms leads to more robust plant growth, especially under stress conditions like drought and heat. It also highlights that a diverse microbial population provides multiple pathways for improving plant health and productivity. For instance, *L. adecarboxylata* and *L. amnigena* have been shown to commonly display multiple plant growth-promoting (PGP) traits such as ACC deaminase activity, and the production of phytohormones (IAA) as well as phosphorus solubilization (Ateş and Kivanç, 2020; El-Akhdar et al., 2020). This synergy between different mechanisms enhances the overall effectiveness of PGPR in promoting plant growth. It is evident that microbes tolerant to a specific abiotic stress can enhance the inferred abiotic tolerance to inoculated plants when exposed to the same stress.

The impact of drought and heat-tolerant PGPR strains on maize seedling growth was studied *in vitro*. Except for *Enterobacter* sp. 14MN3B, all isolates significantly enhanced various growth parameters in maize seedlings subjected to combined drought and heat stress when compared to untreated control plants. *Enterobacter* sp. 14MN3B showed pathogenicity, causing rot on maize plants under the combination of drought and heat stress which was obvious under all replicates (**Figures 2**, **3**). Generally, *Acinetobacter* sp. reportedly has beneficial effects on maize plants. This was the case in *Acinetobacter* sp. S3r2 inoculated maize plants, where a significant increase in the dry biomass and total N content was observed throughout the study (Kuan et al., 2016). Nevertheless, it is worth noting that this is the first instance of maize plants being inoculated with *Acinetobacte*r sp. under the simultaneous influence of drought and heat stress. Consequently, an efficacy evaluation aimed at alleviating dual drought and heat stress in maize under greenhouse conditions was conducted, utilizing only *B. cereus* 11MN1, *B. pseudomyciodes* 21MN1B, *L. amnigena* 33MP1, and *L. adecarboxylata* 36MP8. These isolates were screened for cross antagonism or compatibility using the cross streak dual culturing method. *B. cereus* 11MN1 and *B. pseudomyciodes* 21MN1B demonstrated antimicrobial properties against *L. amnigena* 33MP1, and *L. adecarboxylata* 36MP8 but not against each other (**Supplementary Figure S4**). No antimicrobial properties were observed from *L. amnigena* 33MP1, and *L. adecarboxylata* 36MP8 against the other isolates. This suggests that *B. cereus* 11MN1 and *B. pseudomyciodes* 21MN1B possess antimicrobial properties. Even so, some studies have shown that co-cultures of antagonist microbes can provide still greater plant growth than single inoculations. This was the case with *Trichoderma asperellum* GDFS1009 and *Bacillus amyloliquefaciens* (Karuppiah et al., 2019). *Bacillus amyloliquefaciens* displayed antagonism against *Trichoderma asperellum*, however, seeds treated with the co-culture significantly enhanced the plant growth and protection against phytopathogenic fungi (Karuppiah et al., 2019). Similarly in the current study, despite the antagonism exhibited by *Bacillus* spp. against other isolates, the maize plants still showed enhanced growth under the combined effects of drought and heat stress.

All inoculated plants under combined drought and heat stress showed significant increases in leaf relative water content. The leaf relative water content reflects the balance between water supply to the leaf tissue and transpiration rate. Moreover, it is the most appropriate measure of plant water status in terms of the physiological consequence of cellular water deficit (Teulat et al., 2003; Arndt et al., 2015). In the current study, *B. cereus* 11MN1 and *B. pseudomycoides* 21MN1B could only enhance the leaf RWC among the measured plant growth parameters. Despite no physical changes observed in maize plants inoculated with 11MN1 and 21MN1B, it is likely that changes occurred at the cellular and molecular level. *Lelliotia amnigena* 33MP1 and *L. adecarboxylata* 36MP8 were quite efficient at promoting plant growth under simultaneous drought and heat stress under greenhouse conditions in the study. *Leclercia adecarboxylata* and *L. amnigena* belong to the *Enterobacteriaceae* family, previously called *Escherichia adecarboxylata* and *Enterobacter amnigenus*, respectively. *Lelliottia amnigena* and *L. adecarboxylata* have only recently been discovered as PGPR and have previously been associated with mitigating abiotic stresses (Danish et al., 2020). For instance, one study showed that an ACC deaminase producing *L. adecarboxylata* could alleviate drought stress by improving root elongation (2.2-fold), shoot dry weight (1.5-fold), root dry weight (1.4-fold), NPK uptake, and possibility decreasing ethylene in plants (Notununu et al., 2022a). Teulat et al. (2003) also showed that under osmotic stress, seed inoculation with *L. adecarboxylata* increased root and shoot dry weight, as well as shoot length by 36, 60% and 40.4%, respectively. Likewise, wheat inoculated with ACC deaminase containing *L. adecarboxylata* had significantly improved grain (48.37%) and straw yield (102.13%) under severe drought stress (Notununu et al., 2022a). These studies emphasize the potential of *L. adecarboxylata* and *L. amnigena* at mitigating the effects of abiotic stress. Nevertheless, this shows that treatment with PGPR for the mitigation of abiotic stresses in plants can favor the enhancement of certain measured plant growth parameters over others depending on the PGPR used.

Plant inoculation with a bacterial mixture/consortium has been shown to provide enhanced effects on plant growth in comparison to single bacterial isolate inoculation (Aranda et al., 2012; Lin et al., 2014). In the current study, maize inoculated with the mixture/consortium (11MN1, 21MN1B, 33MP1, 36MP8) performed better than the single isolate inoculation. In general, consortium treatments help plants to maintain growth by accumulating higher biomass even under drought and heat stress conditions. This may be largely attributed to the greater functional diversity resulting from genetic diversity from the different microorganisms that now reside in the rhizosphere. The observed effect can be attributed to specific plant growth-promoting traits of the isolates used in this study. These include their ability to alleviate drought and heat stress through mechanisms like improved water retention and stress resistance, as well as enhancing nutrient availability, particularly phosphorus and nitrogen. Additionally, the production of indole-3-acetic acid (IAA) by these microorganisms stimulates root growth, further improving the plant’s ability to absorb water and nutrients. The functional diversity resulting from the genetic diversity of the microorganisms in the rhizosphere directly supports these processes, leading to the observed positive effects on plant growth. Baig et al. (2012) showed that bacterial isolates with dual PGP traits outperformed isolates with single traits in pot experiments, in terms of increased dry shoot weight (37.6%), root weight (3.9-fold), and root elongation (3.8-fold), as well as grain yield (38.5%). This suggests that the more diverse the PGP traits, the greater the improvements in plant growth.

Under no stress conditions, no significant or apparent increases in plant growth were observed. However, there were significant improvements in RWC for maize plants inoculated with 21MN1B, 33MP1, and the mixture. Likewise, suggesting PGPR-induced changes that occurred at the cellular and molecular levels. Plant inoculation with potential PGPR has been reported to greatly improve yields when evaluated even under ideal climatic situations (de Carvalho et al., 2020). Even so, previous studies have repeatedly shown that the greatest benefits of PGPR occur when crops encounter stressful conditions, especially for prolonged periods (Vardharajula et al., 2010; Egamberdieva et al., 2017). This phenomenon better explains the results obtained in the current study, where significant changes in maize seedlings under stress conditions were observed but not under non-stressed conditions.

To understand the effects of PGPR on the molecular response of plants under these combined stresses, the current study sought to study key stress response genes involved in PGPR-mediated maize tolerance to the effects of dual drought and heat stress, as well as non-stressed conditions. Under non-stressed conditions, all the genes evaluated were downregulated in all treatments. The downregulation of these genes was expected as they play major roles in stress response, and these plants were not stressed. However, under dual drought and heat stress, these genes were differentially expressed in PGPR-inoculated maize. Some of the isolates appear to confer tolerance by modulating the *CAT2* and *DHN2* stress response genes. Catalase activity is concomitantly increased in plants as a manifestation of the adaptive response of plants to drought and heat stress (Sofo et al., 2015; Laxa et al., 2019). Plant growth-promoting rhizobacteria have been reported to induce tolerance to plants through enhanced modulation of the catalase enzyme. For instance, *Pseudomonas* application on maize under severe water stress increased the CAT activity by 50% compared to well-watered plants, alleviating the adverse effects of drought stress on the physiological characteristics of maize (Rezazadeh et al., 2019). In the present study, *DHN2* expression varied with each PGPR-inoculated maize, however, was significantly decreased in plants inoculated with the mixture of all isolates. Dehydrin proteins bind to partly dehydrated surfaces of proteins, protecting them from protein denaturation, and may also exhibit ROS scavenging properties (Shih et al., 2008; Xing et al., 2011). The mixture of all isolates which recorded the greatest in root and shoot dry biomass may have reduced the dehydration effect by some other mechanism, hence there was no need for upregulation of the *DHN2* gene. This phenomenon is also observed with the *HSP70* transcript. The *HSP70* gene was downregulated in all maize treatments under the dual stress conditions, except those inoculated with 21MN1B.

Overall, the results obtained on the stress response of genes under concurrent drought and heat stress support the suggested strain-specific molecular responses of plants to PGPR inoculation (Drogue et al., 2012). It has also been suggested that PGPR-mediated plant growth is a multigenic process that may be specific to the participating PGPR and plant species (Bharti et al., 2016). Consortium treatments aid plants in sustaining growth under drought and heat stress due to increased functional diversity due to the higher functional diversity, possibly contributing multiple roles in plant growth promotion, osmoprotection, thermotolerance, and antioxidant activity, resulting in stress alleviation. Furthermore, the differential expression of *CAT2*, *DHN2*, and *HSP70* observed in maize plants under concurrent drought and heat stress suggests this specificity between PGPR and plant species.

## 5 Conclusion

This study identified PGPR isolates suitable for inclusion in biofertilizers designed to enhance plant adaptation to drought and heat stress. The results highlight the potential of bacterial isolates *B. cereus* 11MN1, *B. pseudomycoides* 21MN1B, *L. amnigena* 33MP1, and *L. adecarboxylata* 36MP8 in promoting maize growth under dual drought and heat stress. Specifically, *L. amnigena* 33MP1, *L. adecarboxylata* 36MP8, and the combined use of all isolates demonstrated the most promising ability to confer tolerance in maize under these challenging conditions. These PGPR strains appear to induce tolerance by initiating Induced Systemic Tolerance and by modulating the *CAT2* and *DHN2* stress response genes. However, further research is necessary to fully understand the molecular changes induced by these PGPRs in maize plants under simultaneous drought and heat stress. This study introduces an alternative natural approach to significantly enhance maize production in the face of adverse climate conditions, benefiting both commercial and small-scale farmers. The application of these drought and heat tolerant PGPR in agriculture offers an opportunity to reduce the reliance on extensive irrigation and chemical fertilizers throughout the maize crop’s growth cycle. Given the ongoing global climate change challenges and the resulting decreased crop yields, the findings of this study have the potential to boost the productivity of communities using traditional agricultural methods.

## Data Availability

The datasets presented in this study can be found in online repositories. The names of the repository/repositories and accession number(s) can be found in the article/**Supplementary Material**.
