# Supplementary material for: Enhancing maize drought and heat tolerance: single vs combined plant growth promoting rhizobacterial inoculation
Source: Front Plant Sci. 2024 Dec 10;15:1480718. doi: 10.3389/fpls.2024.1480718 (PMC11667205; doi:10.3389/fpls.2024.1480718)
Supplement: Supplementary file 1 [file DataSheet1.pdf]

Supplementary information

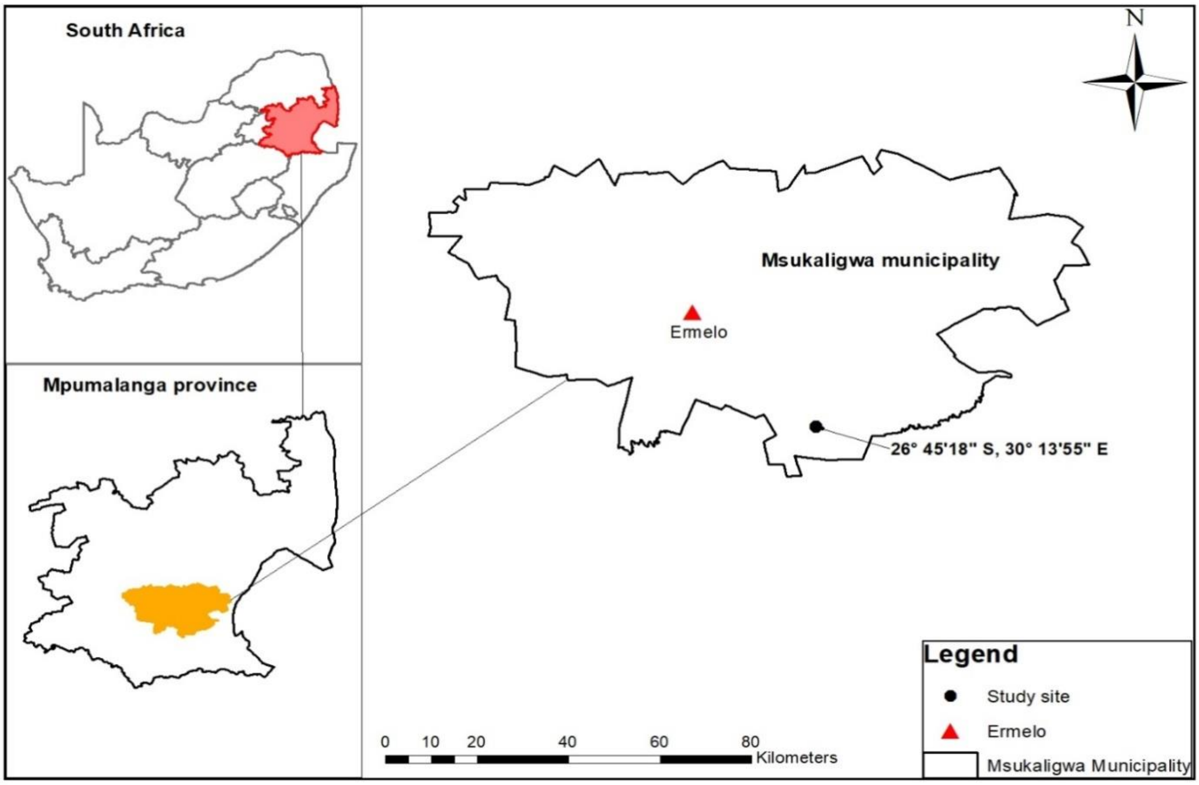

Supplementary Fig. S1: The study site in Mpumalanga Province of South Africa

Supplementary Table S1: Candidate stress response genes and the reference genes, their primer sequences and product sizes.

Gene Name	Primer Name	Primer Sequence	Product size	Reference
**Dehydrin 2**	dhn2-624F	5’-ACGAAGACTCAGACCCCACCA-3’	104	(Capelle *et al*., 2010)
	dhn2-727R	5’-GCGTCTTCCGGCTTCTTGT-3’		
**Heat Shock Protein 70**	Hps70F	5’-AGCTAAGACTGGGTGGCTGA- 3’		Designed
	Hsp70R	5’-GTCGTCTTCTCCCTGTGCTC-3’		
**Catalase 2**	Cat2_F	5’-TCTCTGTCTGCTTTCGCTCA-3’	151	Designed
	Cat2_R	5’-GGACACAGCCAGCCATTATT-3’		

**Tubulin beta (β-Tub)**	β-TUB_F	5'- CTACCTCACGGCATCTGCTATGT-3'	139	Lin *et al.*, 2014
	β-TUB_R	5'- GTCACACACACTCGACTTCACG-3'		
**Elongation factor 1 alpha (EF1a)**	EF1a_F	5'- TGGGCCTACTGGTCTTACTACTGA-3'	135	Lin *et al.*, 2014
	EF1a_R	5'- ACATACCCACGCTTCAGATCCT-3'		

Supplementary Table S2: Screened bacterial isolates for drought (40 % PEG 6000) and heat stress (42 °C) cultured on TSB broth. Bacterial isolates that exhibited an OD above 0.40 for both drought and heat stress were considered drought and heat tolerant. Bacterial isolates coloured green exhibited tolerance to both drought and heat stress

Sample No:	SAMPLE ID	Heat stress		Drought stress	
		Average Optical density (OD)	Standard Deviation	Average Optical density (OD)	Standard Deviation
1	21MN1A	1.09	0.04	0.08	0.18
2	32MN1B	1.10	0.01	0.50	0.09
3	15MN6B	0.71	0.04	0.16	0.05
4	15MN5	1.34	0.10	0.10	0.13
5	32MN2B	1.23	0.10	0.03	0.04
6	14MN3B	1.05	0.17	1.15	0.90
7	22MN2A	0.87	0.11	0.02	0.05
8	14MN3A	1.00	0.10	0.38	0.22
9	14MN5A	1.12	0.00	0.49	0.10

10	14MN5B	0.92	0.08	0.01	0.03
11	22MN2B	1.02	0.03	0.45	0.04
12	11MN3	0.93	0.01	0.42	0.06
13	21MN1B	0.94	0.14	0.45	0.14
14	14MN5A	0.27	—	0.45	0.10
15	11MN1	1.07	0.04	0.53	0.06
16	15MN6B	0.70	0.04	0.15	0.05
17	32MN1A	1.07	0.04	0.37	0.10
18	21MN3	1.00	0.01	0.35	0.10
19	11MN2	1.14	0.04	0.50	0.08
20	32MN3	0.95	0.02	0.37	0.07
21	32MN4	1.10	0.04	0.10	0.06
22	14MN1	1.09	0.01	0.28	0.17
23	22MN2	0.90	0.04	0.33	0.08
24	31MN1B	0.66	0.04	0.50	0.19
25	21MN3S	1.16	0.06	0.34	0.07
26	35MN3	0.89	0.03	0.37	0.14
27	31MN1S	1.30	0.02	0.12	0.02
28	23MN5	0.91	0.03	0.37	0.08
29	14MN2	1.12	0.03	0.24	0.15
30	35MN1	0.85	0.04	0.10	0.09
31	26MP3	1.47	0.08	0.03	0.09
32	20MP2S	1.24	0.03	0.00	—

33	27MP1	0.11	0.03	0.03	0.08
34	33MP1	0.87	0.04	0.54	0.21
35	15MP4	1.00	0.06	-0.06	0.03
36	14MP3	1.06	0.02	0.29	0.06
37	26MP3	1.07	0.01	0.08	0.06
38	30MP4	1.15	0.09	0.14	0.10
39	16MP1	0.87	0.01	0.04	0.16
40	15MP2	0.18	0.04	0.01	0.04
41	36MP8	0.64	0.01	0.96	0.30
42	14MP4	1.22	0.15	0.07	0.04
43	20MP2B	1.14	0.19	0.21	0.14
44	18MP2	1.20	0.05	0.10	0.09
45	12MP2	1.04	0.05	-0.02	0.06
46	23MP1	1.13	0.05	0.16	0.08
47	36MP1	0.74	0.05	0.40	0.20
48	26MP4Y	0.87	0.08	0.22	0.11
49	28MP1W	0.37	0.04	0.14	0.03
50	30MP5	1.00	0.03	0.37	0.05
51	31MP1	0.59	0.04	0.06	0.10
52	30MP3Y	0.93	0.03	0.18	0.03
53	23MP3	0.29	0.07	0.03	0.03
54	34MP2	0.86	0.05	0.48	0.11
55	36MP4	0.89	0.03	0.33	0.12
56	26MP2	0.91	0.03	0.14	0.09
57	21MP2Y	0.01	__	0.14	0.05
58	19MP4Y	0.77	0.41	-0.02	__

59	21MP1	1.08	0.01	0.21	0.04
60	21MP2	-0.08	__	-0.03	0.01
61	19MP4 W	0.27	0.02	-0.02	0.00

Supplementary Table S3*:* 16S rDNA gene sequence similarity (%) of test isolates to known isolates

Isolate ID	Sequence analysis results	% Similarity
**36MP8**	***Leclercia_sp._strain_T3196-2***	**100**
**34MP2**	***Leclercia_sp._strain_T3196-2***	**99**
**33MP1**	***Lelliottia_amnigena_strain_NCTC12124***	**99**
**32MN1B**	***Bacillus_cereus _ strain 24195***	**100**
**31MN1**	***Bacillus_ cereus _ strain 24195***	**100**
**21MN2**	***Bacillus_ cereus _ strain 24195***	**100**
**21MN1A**	***Bacillus_pseudomycoides_strain_MF-68***	**100**
**14MN5A**	***Bacillus_ cereus _ strain 24195***	**100**
**14MN3B**	***Acinetobacter_sp.*_DSM30007**	**99**
**11MN3**	***Bacillus_ cereus _ strain 24195***	**100**
**11MN2**	***Bacillus_ cereus _ strain 24195***	**100**
**11MN1**	***Bacillus_ cereus _ strain 24195***	**100**

Sequence data for species identification

>32MN1B

GCGTGAGTGATGAAGGCTTTCGGGTCGTAAAACTCTGTTGTTAGGGAAGAACAAGTGCTA

GTTGAATAAGCTGGCACCTTGACGGTACCTAACCAGAAAGCCACGGCTAACTACGTGCCA

GCAGCCGCGGTAATACGTAGGTGGCAAGCGTTATCCGGAATTATTGGGCGTAAAGCGCGC

GCAGGTGGTTTCTTAAGTCTGATGTGAAAGCCCACGGCTCAACCGTGGAGGGTCATTGGA

AACTGGGAGACTTGAGTGCAGAAGAGGAAAGTGGAATTCCATGTGTAGCGGTGAAATGCG

TAGAGATATGGAGGAACACCAGTGGCGAAGGCGACTTTCTGGTCTGTAACTGACACTGAG

GCGCGAAAGCGTGGGGAGCAAACAGGATTAGATACCCTGGTAGTCCACGCCGTAAACGAT

GAGTGCTAAGTGTTAGAGGGTTTCCGCCCTTTAGTGCTGAAGTTAACGCATTAAGCACTC

CGCCTGGGGAGTACGGCCGCAAGGCTGAAACTC

>11MN1

GCCGCGTGAGTGATGAAGGCTTTCGGGTCGTAAAACTCTGTTGTTAGGGAAGAACAAGTG

CTAGTTGAATAAGCTGGCACCTTGACGGTACCTAACCAGAAAGCCACGGCTAACTACGTG

CCAGCAGCCGCGGTAATACGTAGGTGGCAAGCGTTATCCGGAATTATTGGGCGTAAAGCG

CGCGCAGGTGGTTTCTTAAGTCTGATGTGAAAGCCCACGGCTCAACCGTGGAGGGTCATT

GGAAACTGGGAGACTTGAGTGCAGAAGAGGAAAGTGGAATTCCATGTGTAGCGGTGAAAT

GCGTAGAGATATGGAGGAACACCAGTGGCGAAGGCGACTTTCTGGTCTGTAACTGACACT

GAGGCGCGAAAGCGTGGGGAGCAAACAGGATTAGATACCCTGGTAGTCCACGCCGTAAAC

GATGAGTGCTAAGTGTTAGAGGGTTTCCGCCCTTTAGTGCTGAAGTTAACGCATTAAGCA

CTCCGCCTGGGGAGTACGGCCGCAAGG

>11MN2

GCTTTCGGGTCGTAAAACTCTGTTGTTAGGGAAGAACAAGTGCTAGTTGAATAAGCTGGC

ACCTTGACGGTACCTAACCAGAAAGCCACGGCTAACTACGTGCCAGCAGCCGCGGTAATA

CGTAGGTGGCAAGCGTTATCCGGAATTATTGGGCGTAAAGCGCGCGCAGGTGGTTTCTTA

AGTCTGATGTGAAAGCCCACGGCTCAACCGTGGAGGGTCATTGGAAACTGGGAGACTTGA

GTGCAGAAGAGGAAAGTGGAATTCCATGTGTAGCGGTGAAATGCGTAGAGATATGGAGGA

ACACCAGTGGCGAAGGCGACTTTCTGGTCTGTAACTGACACTGAGGCGCGAAAGCGTGGG

GAGCAAACAGGATTAGATACCCTGGTAGTCCACGCCGTAAACGATGAGTGCTAAGTGTTA

GAGGGTTTCCGCCCTTTAGTGCTGAAGTTAACGCATTAAGCACTCCGCCTGGGGAGTACG

>11MN3

GTGATGAAGGCTTTCGGGTCGTAAAACTCTGTTGTTAGGGAAGAACAAGTGCTAGTTGAA

TAAGCTGGCACCTTGACGGTACCTAACCAGAAAGCCACGGCTAACTACGTGCCAGCAGCC

GCGGTAATACGTAGGTGGCAAGCGTTATCCGGAATTATTGGGCGTAAAGCGCGCGCAGGT

GGTTTCTTAAGTCTGATGTGAAAGCCCACGGCTCAACCGTGGAGGGTCATTGGAAACTGG

GAGACTTGAGTGCAGAAGAGGAAAGTGGAATTCCATGTGTAGCGGTGAAATGCGTAGAGA

TATGGAGGAACACCAGTGGCGAAGGCGACTTTCTGGTCTGTAACTGACACTGAGGCGCGA

AAGCGTGGGGAGCAAACAGGATTAGATACCCTGGTAGTCCACGCCGTAAACGATGAGTGC

TAAGTGTTAGAGGGTTTCCGCCCTTTAGTGCTGAAGTTAACGCATTAAGCACTCCGCCTG

GGGAGTAC

>21MN2B

GcCGCGTGAGTGATGAAGGCTTTCGGGTCGTAAAACTCTGTtGTTaGGGaAGAACAAGTG

CTAGTTGAATAAGCTGGCACCTTGACGGTACCTAACCAGAAAGCCACGGCTAACTACGTG

CCAGCAGCCGCGGTAATACGTAGGTGGCAAGCGTTATCCGGAATTATTGGGCGTAAAGCG

CGCGCAGGTGGTTTCTTAAGTCTGATGTGAAAGCCCACGGCTCAACCGTGGAGGGTCATT

GGAAACTGGGAGACTTGAGTGCAGAAGAGGAAAGTGGAATTCCATGTGTAGCGGTGAAAT

GCGTAGAGATATGGAGGAACACCAGTGGCGAAGGCGACTTTCTGGTCTGTAACTGACACT

GAGGCGCGAAAGCGTGGGGAGCAAACAGGATTAGATACCCTGGTAGTCCACGCCGTAAAC

GATGAGTGCTAAGTGTTAGAGGGTTTCCGCCCTTTAGTGCTGAAGTTAACGCATTAAGCA

CTCCGCCTGGGGAGTACGGCCGCAAGGCTGAAACTCAAAGG

>31MN1B

GCCGCGTGAGTGATGAAGGCTTTCGGGTCGTAAAACTCTGTTGTTAGGGAAGAACAAGTG

CTAGTTGAATAAGCTGGCACCTTGACGGTACCTAACCAGAAAGCCACGGCTAACTACGTG

CCAGCAGCCGCGGTAATACGTAGGTGGCAAGCGTTATCCGGAATTATTGGGCGTAAAGCG

CGCGCAGGTGGTTTCTTAAGTCTGATGTGAAAGCCCACGGCTCAACCGTGGAGGGTCATT

GGAAACTGGGAGACTTGAGTGCAGAAGAGGAAAGTGGAATTCCATGTGTAGCGGTGAAAT

GCGTAGAGATATGGAGGAACACCAGTGGCGAAGGCGACTTTCTGGTCTGTAACTGACACT

GAGGCGCGAAAGCGTGGGGAGCAAACAGGATTAGATACCCTGGTAGTCCACGCCGTAAAC

GATGAGTGCTAAGTGTTAGAGGGTTTCCGCCCTTTAGTGCTGAAGTTAACGCATTAAGCA

CTCCGCCTGGGGAGTACGGCCGCAAGGCTGAAACTCAAAG

>14MN5A

CGTGAGTGATGAAGGCTTTCGGGTCGTAAAACTCTGTTGTTAGGGAAGAACAAGTGCTAG

TTGAATAAGCTGGCACCTTGACGGTACCTAACCAGAAAGCCACGGCTAACTACGTGCCAG

CAGCCGCGGTAATACGTAGGTGGCAAGCGTTATCCGGAATTATTGGGCGTAAAGCGCGCG

CAGGTGGTTTCTTAAGTCTGATGTGAAAGCCCACGGCTCAACCGTGGAGGGTCATTGGAA

ACTGGGAGACTTGAGTGCAGAAGAGGAAAGTGGAATTCCATGTGTAGCGGTGAAATGCGT

AGAGATATGGAGGAACACCAGTGGCGAAGGCGACTTTCTGGTCTGTAACTGACACTGAGG

CGCGAAAGCGTGGGGAGCAAACAGGATTAGATACCCTGGTAGTCCACGCCGTAAACGATG

AGTGCTAAGTGTTAGAGGGTTTCCGCCCTTTAGTGCTGAAGTTAACGCATTAAGCACTCC

GCCTGGGGAGTACGGCCGCAAGGCTGAAACTC

>21MN1B

AAAgCTCTGTtGtTAGGGAAGAACAAGTGCTAGTTGAATAAGCTGGCACCTTGACGGTAC

CTAACCAGAAAGCCACGGCTAACTACGTGCCAGCAGCCGCGGTAATACGTAGGTGGCAAG

CGTTATCCGGAATTATTGGGCGTAAAGCGCGCGCAGGTGGTTTCTTAAGTCTGATGTGAA

AGCCCACGGCTCAACCGTGGAGGGTCATTGGAAACTGGGAGACTTGAGTGCAGAAGAGGA

AAGTGGAATTCCATGTGTAGCGGTGAAATGCGTAGAGATATGGAGGAACACCAGTGGCGA

AGGCGACTTTCTGGTCTGTAACTGACACTGAGGCGCGAAAGCGTGGGGAGCAAACAGGAT

TAGATACCCTGGTAGTCCACGCCGTAAACGATGAGTGCTAAGTGTTAGAGGGTTTCCGCC

CTTTAGTGCTGAAGTTAACGCATTAAGCACTCCGCCTGGGGAGTACGGCCGCAAGGCTGA

AAC

>14MN3B

GCGTGTGTGAGAAGGCCTTATGGTTGTAAAGCACTTTAAGCGAGGAGGAGGCTACcTAGT

TAATACCTAGgGATAGTGGACGTTACTCGCAGAATAAGCACCGGCTAACTCTGTGCCAGC

AGCCGCGGTAATACAGAGGGTGCGAGCGTTAATCGGATTTACTGGGCGTAAAGCGTGCGT

AGGCGGCTTATTAAGTCGGATGTGAAATCCCCGAGCTTAACTTGGGAATTGCATTCGATA

CTGGTGAGCTAGAGTATGGGAGAGGATGGTAGAATTCCAGGTGTAGCGGTGAAATGCGTA

GAGATCTGGAGGAATACCGATGGCGAAGGCAGCCATCTGGCCTAATACTGACGCTGAGGT

ACGAAAGCATGGGGAGCAAACAGGATTAGATACCCTGGTAGTCCATGCCGTAAACGATGT

CTACTAGCCGTTGGGGCCTTTGAGGCTTTAGTGGCGCAGCTAACGCGATAAGTAGACCGC

CT

>33MP1

TGCCGCGTGTATGAAGAAGGCCTTCGGGTTGTAAAGTACTTTCAGCGAGGAGGAAGGCgT

TGtGGTTAATAACCaCAgtGATTGACGTTACTCGCAGAAGAAGCACCGGCTAACTCCGTG

CCAGCAGCCGCGGTAATACGGAGGGTGCAAGCGTTAATCGGAATTACTGGGCGTAAAGCG

CACGCAGGCGGTCTGTCAAGTCGGATGTGAAATCCCCGGGCTCAACCTGGGAACTGCATT

CGAAACTGGCAGGCTAGAGTCTTGTAGAGGGGGGTAGAATTCCAGGTGTAGCGGTGAAAT

GCGTAGAGATCTGGAGGAATACCGGTGGCGAAGGCGGCCCCCTGGACAAAGACTGACGCT

CAGGTGCGAAAGCGTGGGGAGCAAACAGGATTAGATACCCTGGTAGTCCACGCCGTAAAC

GATGTCGACTTGGAGGTTGTTCCCTTGAGGAGTGGCTTCCGGAGCTAACGCGTTAAGTCG

ACCGCCTGGGGAGTACGGCCGCAA

>34MP2

TGCCGCGTGTATGAAGAAGGCCTTCGGGTTGTAAAGTACTTTCAGCGGGGAGGAAGGTGT

TGtGGTTAATAACCGCAGCAATTGACGTTACCCGCAGAAGAAGCACCGGCTAACTCCGTG

CCAGCAGCCGCGGTAATACGGAGGGTGCAAGCGTTAATCGGAATTACTGGGCGTAAAGCG

CACGCAGGCGGTCTGTCAAGTCGGATGTGAAATCCCCGGGCTCAACCTGGGAACTGCATT

CGAAACTGGCAGGCTAGAGTCTTGTAGAGGGGGGTAGAATTCCAGGTGTAGCGGTGAAAT

GCGTAGAGATCTGGAGGAATACCGGTGGCGAAGGCGGCCCCCTGGACAAAGACTGACGCT

CAGGTGCGAAAGCGTGGGGAGCAAACAGGATTAGATACCCTGGTAGTCCACGCCGTAAAC

GATGTCGACTTGGAGGTTGTTCCCTTGAGGAGTGGCTTCCGGAGCTAACGCGTTAAGTCG

ACCGCCTGGGGAGTACGGCCGC

>36MP8

TGCCGCGTGTATGAAGAAGGCCTTCGGGTTGTAAAGTACTTTCAGCGgGGAGGAAGGTGT

TGtGGTTAATAACCgCAGCaATTGACGTTACcCGCAGAAGAAGCACCGGCTAACTCCGTG

CCAGCAGCCGCGGTAATACGGAGGGTGCAAGCGTTAATCGGAATTACTGGGCGTAAAGCG

CACGCAGGCGGTCTGTCAAGTCGGATGTGAAATCCCCGGGCTCAACCTGGGAACTGCATT

CGAAACTGGCAGGCTAGAGTCTTGTAGAGGGGGGTAGAATTCCAGGTGTAGCGGTGAAAT

GCGTAGAGATCTGGAGGAATACCGGTGGCGAAGGCGGCCCCCTGGACAAAGACTGACGCT

CAGGTGCGAAAGCGTGGGGAGCAAACAGGATTAGATACCCTGGTAGTCCACGCCGTAAAC

GATGTCGACTTGGAGGTTGTTCCCTTGAGGAGTGGCTTCCGGAGCTAACGCGTTAAG

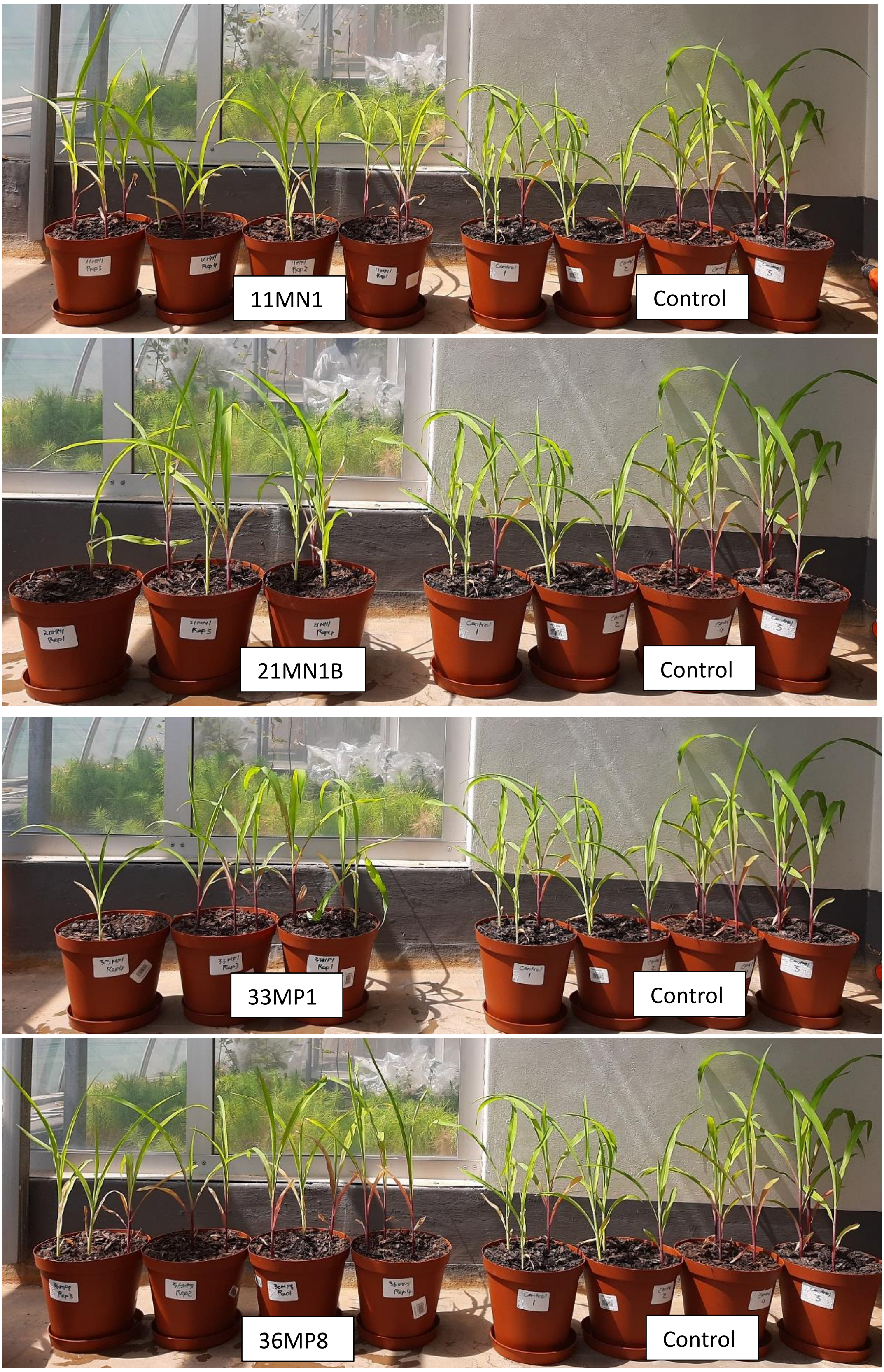
11MN1
Control
21MN1B
Control
33MP1
Control
36MP8
Control

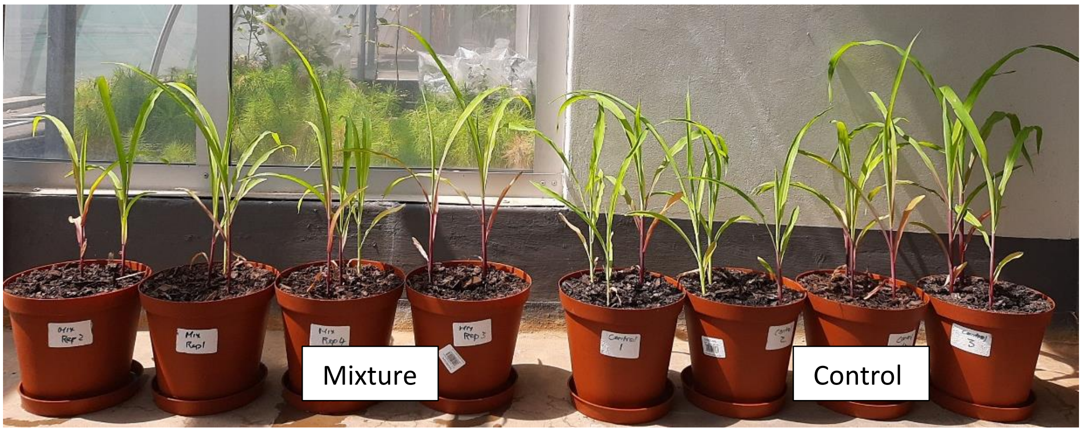

Supplementary Fig. S2: Visual depiction of bacterized maize seeds grown under ambient conditions with soil moisture maintained at 80 % WHC and the temperature at 25/23 °C after 32 days.

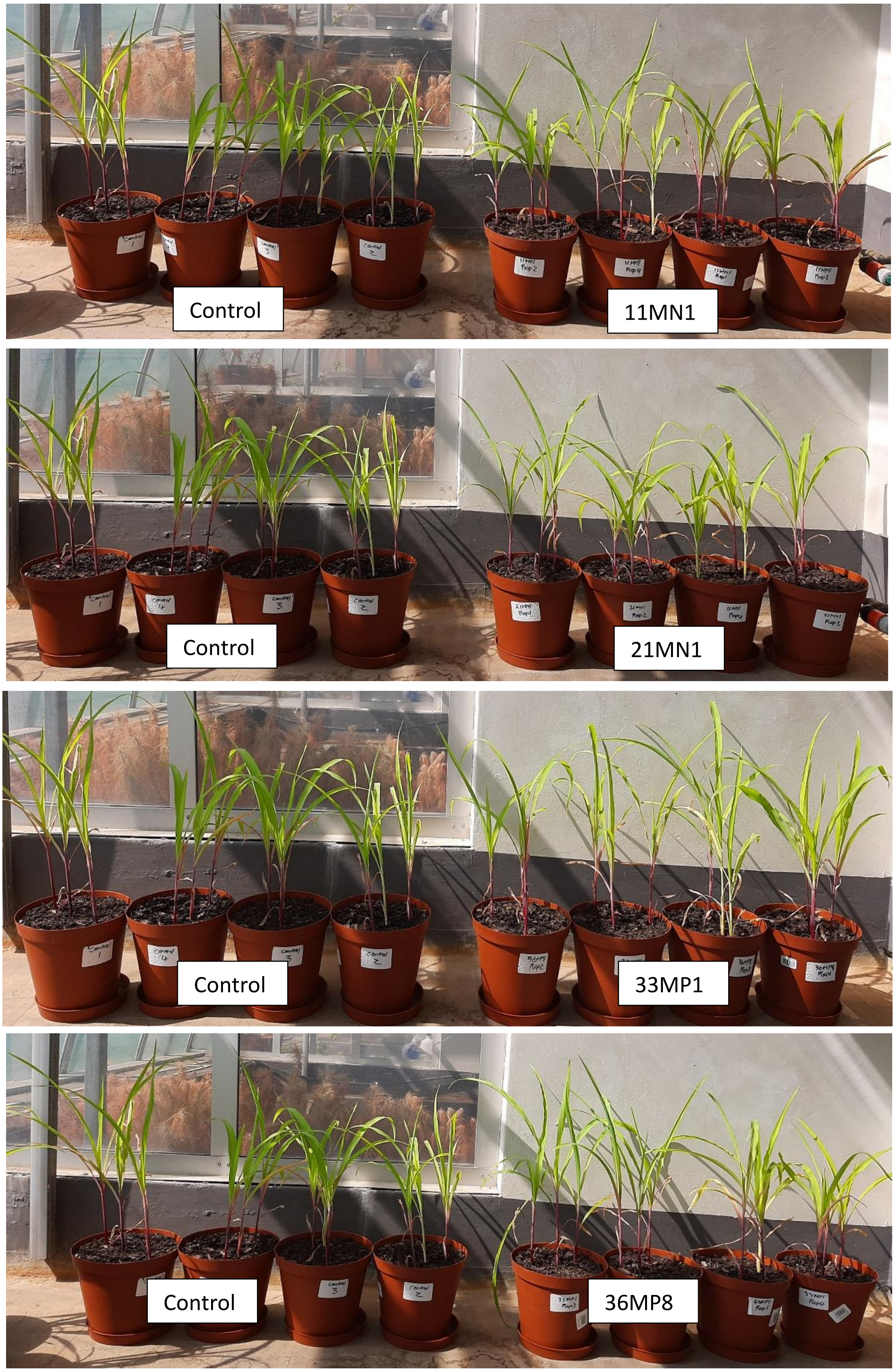
Control
11MN1
Control
21MN1
Control
33MP1
Control
36MP8

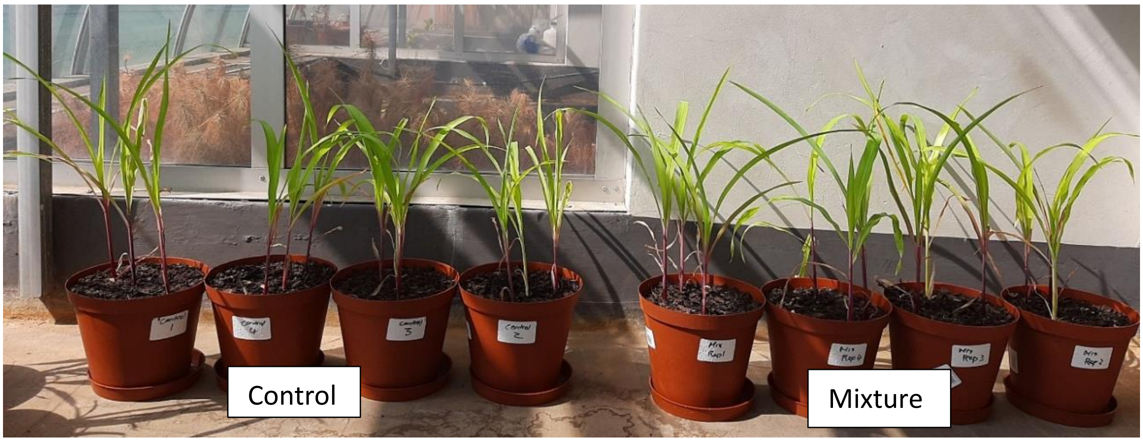

Supplementary Fig. S3: Visual depiction of bacterized maize seeds grown under the concurrent stress of drought (40 % WHC) and heat (32/28 °C) stress after 32 days.
